# 5-Formyltriazoles as Valuable Starting Materials for Unsymmetrically Substituted Bi-1,2,3-Triazoles

**DOI:** 10.3389/fchem.2020.00271

**Published:** 2020-04-15

**Authors:** Robby Vroemans, Tomas Horsten, Maarten Van Espen, Wim Dehaen

**Affiliations:** Molecular Design and Synthesis, Department of Chemistry, KU Leuven, Leuven, Belgium

**Keywords:** bi-1,2,3-triazole, atropisomer, nitro compounds, 1,2,3-triazoles, 1,3-dipolar cycloadditions

## Abstract

Herein, we present the first synthetic methodologies toward non-symmetrical 5,5′-*C, C*-linked bi-1,2,3-triazoles starting from 5-formyl-1,2,3-triazole *via* two related pathways. In a first reaction, 5-formyl-1,2,3-triazole is successfully reacted with a variety of nitroalkanes and organic azides in a one-pot three-component fashion resulting in tetra-*ortho*-substituted bi-1,2,3-triazoles. In the second, closely related reaction, 5-formyl-1,2,3-triazole is initially converted with nitromethane to the corresponding nitroalkene, and then subsequently oxidatively cyclized with a number of organic azides toward 4-nitro substituted non-symmetrical tetra-*ortho*-substituted 5,5′-bi-1,2,3-triazoles. The scope of both reactions and furtherr post-functionalizations are examined, and the atropisomeric properties of the obtained bi-1,2,3-triazoles are evaluated.

## Introduction

The synthesis of bi-1,2,3-triazole derivatives is scarcely reported, and hence their characteristics and potential applications in e.g., enantioselective synthesis, are strongly underexplored (Zheng et al., 2015; Dawood et al., 2018). The bi-1,2,3-triazoles that have been reported to date are divided into three groups in recent reviews, i.e., symmetrical and unsymmetrical 4,4′-bi-1,2,3-triazoles, and symmetrical 5,5'-bi-1,2,3-triazoles (Zheng et al., 2015; Dawood et al., 2018). For the latter type, the only used method is a modified copper(I)-catalyzed azide-alkyne cycloaddition reaction first reported by Burgess in 2007 (Angell and Burgess, 2007). *Via* this way, only symmetrical bi-1,2,3-triazoles can be obtained (Angell and Burgess, 2007; Oladeinde et al., 2010; González et al., 2011; Kwon et al., 2012; Zheng et al., 2012, 2015; Wang et al., 2014; Hoyo et al., 2015; Brassard et al., 2016; Etayo et al., 2017; Dawood et al., 2018; Li et al., 2018; Singh et al., 2018). Interestingly, Péricas et al. reported the preparation of 5,5′-bi-1,2,3-triazoles derived from an enantiopure and sterically encumbered propargylamine, but the target compound was only obtained in a poor yield of 8% (Etayo et al., 2017). These 5,5′-bi-1,2,3-triazoles exhibited a relatively high conformational stability (113–117 kJ/mol at 75°C in toluene-*d*_8_), and were successfully applied in the scandium-catalyzed addition of indoles to isatin. Other axially chiral *N, N*-dimethylpropargylamine-derived bi-1,2,3-triazoles showed fast rotation at room temperature (79.5 kJ/mol at 25°C in n-hexane/ethanol) (Etayo et al., 2017). This was the first study of the conformational stability of 5,5′-bi-1,2,3-triazoles. Nonetheless, this field remains strongly underexplored. The lack of investigation in this area could be ascribed to the complicated syntheses of both starting materials (functionalized alkynes) and bi-1,2,3-triazoles themselves, and hence the cumbersome introduction of functional groups interesting for various applications (Zheng et al., 2015; Dawood et al., 2018). In regard with our current interests in sterically encumbered fully substituted (atropisomeric) 1,2,3-triazoles (Thomas et al., 2014, 2016; Vroemans et al., 2018; Krasniqi and Dehaen, 2019), we report what are to the best of our knowledge the first pathways toward novel unsymmetrically tetra-*ortho*-substituted 5,5′-bi-1,2,3-triazoles from easily accessible starting materials. The anticipated highly sterically hindered bi-1,2,3-triazoles could be decorated with various functional groups which can be introduced in a straightforward manner from *ortho, ortho*'(1,4-)-disubstituted 5-formyl-1,2,3-triazoles, and nitroalkane derivatives.

## Results and Discussion

**Scheme 1:**
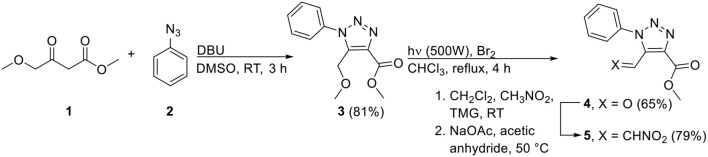
Synthesis of 5-formyl- and 5-nitrovinyl-appended 1,2,3-triazoles **4** and **5** (TMG = 1,1,3,3-tetramethylguanidine).

The common precursor that was envisaged for the synthesis of the desired tetra-*ortho*-substituted bi-1,2,3-triazoles **8** and **9** is 5-formyl-1,2,3-triazole **4**, which itself can be easily prepared either from the commercially available methyl 4-methoxyacetoacetate **1** (Scheme 1; NMR spectra of all novel compounds are displayed in the Supplementary Material) or ethyl 4,4-diethoxy-3-oxobutanoate (L'abbé and Dehaen, 1988; Pokhodylo et al., 2018). The aldehyde moiety serves as a versatile tool which can be employed in our in-house developed three-component reaction (Thomas et al., 2014), or can be converted to nitroalkene derivative **5** (Nomland and Hills, 2008) which can subsequently undergo the copper-catalyzed oxidative [3+2]-cycloaddition reaction reported by the group of Chen (Chen et al., 2015). Hence, we commenced by synthesizing starting materials **4** and **5**, which was initiated by performing the Dimroth reaction with methyl 4-methoxyacetoacetate **1** and phenyl azide **2**, and not starting from ethyl 4,4-diethoxy-3-oxobutanoate which needs to be prepared itself. The desired 5-methoxybenzyl analog **3** was easily obtained on multigram scale *via* subsequent precipitation and recrystallization. Next, aldehyde **4** was obtained *via* the photochemical conversion of the methyl ether with bromine in 65% yield. For the second procedure, i.e., the oxidative [3+2]-cycloaddition reaction, 5-formyl-1,2,3-triazole **4** was converted into nitroalkene derivative **5**. With both aldehyde **4** and nitroalkene **5** available on gram scale, these could now be employed as starting materials in the subsequent synthesis of unsymmetrical tetra-*ortho*-substituted 5,5′-bi-1,2,3-triazoles **8** and **9**.

**Figure 1:**
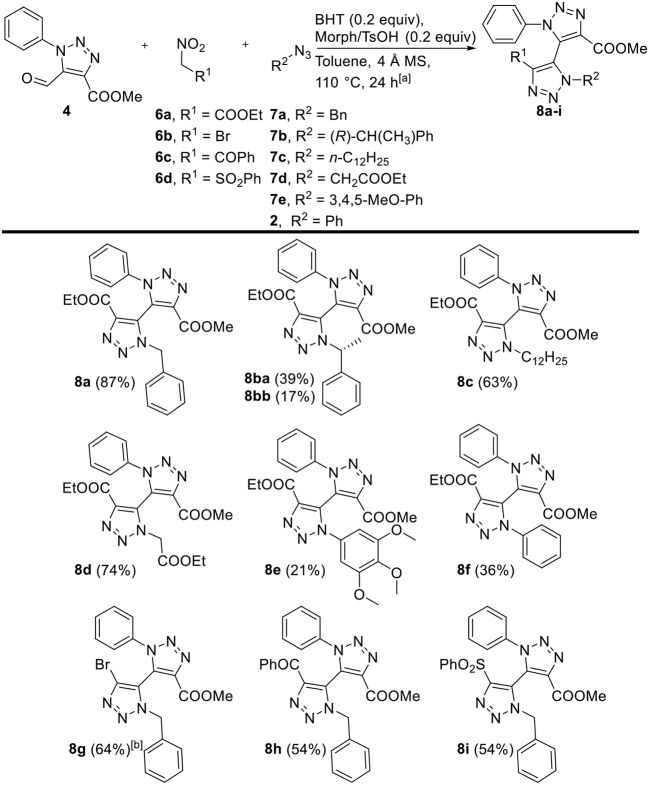
Scope of three-component reaction toward tetra-*ortho*-substituted 5,5'-bi-1,2,3-triazoles **8a-i**, variations with respect to 5-formyl-1,2,3-triazole **4**, nitroalkanes **6a–d**, and organic azides **2**, **7a–e**. ^[a]^ Reaction conditions: **4** (1 equiv), nitroalkane **6a**–**d** (1.3 equiv), organic azide **2**, **7a**–**e** (2 equiv), BHT (0.2 equiv), Morph/TsOH (0.2 equiv), 4 Å MS (50 mg), dry toluene (0.2 mL), 110°C. ^[b]^ Reaction conditions: **4** (1 equiv), bromonitromethane (1.2 equiv), TMG (0.1 equiv), dry DCM (0.4 mL); then, NaOAc (1 equiv), acetic anhydride (10 equiv), 50°C; then, **7a** (1.5 equiv), BHT (0.1 equiv) and PTSA·H_2_O (0.1 equiv), dry toluene (1 mL), 110°C.

Next, the three-component reaction was applied as described by our group on 5-formyl-1,2,3-triazole **4** with a variety of nitroalkanes **6a-d** and organic azides **2**, **7a-e** (Figure 1). Firstly, various alkyl and aryl azides were employed with aldehyde **4** and ethyl nitroacetate **6a** as model substrates. Good to excellent yields (56–87%) of bi-1,2,3-triazoles **8** were obtained for alkyl azides **7a**-**d**. Interestingly, enantiopure chiral alkyl azide (*R*)-**7b** yielded a 2.25:1-diastereomeric mixture, which was successfully separated *via* silica gel chromatography into single diastereomers **8ba** and **8bb**. In general, the use of aryl azides **2** and **7e** involved an extended reaction time and slightly decreased yields (21–36%) compared to alkyl azides, and the reaction even failed to afford any product when electron-deficient 4-nitrophenyl azide was employed. Secondly, other functional groups were introduced by varying nitroalkanes **6a**-**6d**. Benzoyl- and phenylsulfonyl-appended bi-1,2,3-triazoles **8h** and **8i**, respectively, were both prepared in 54% yield. The direct three-component reaction toward bromo-derivative **8g** seemed cumbersome, in which the formation of the intermediate bromonitroalkene did not proceed. Nevertheless, **8g** was isolated in 64% *via* a two-pot procedure without intermediate purification of the *in situ* generated bromonitroalkene derivative.

**Figure 2:**
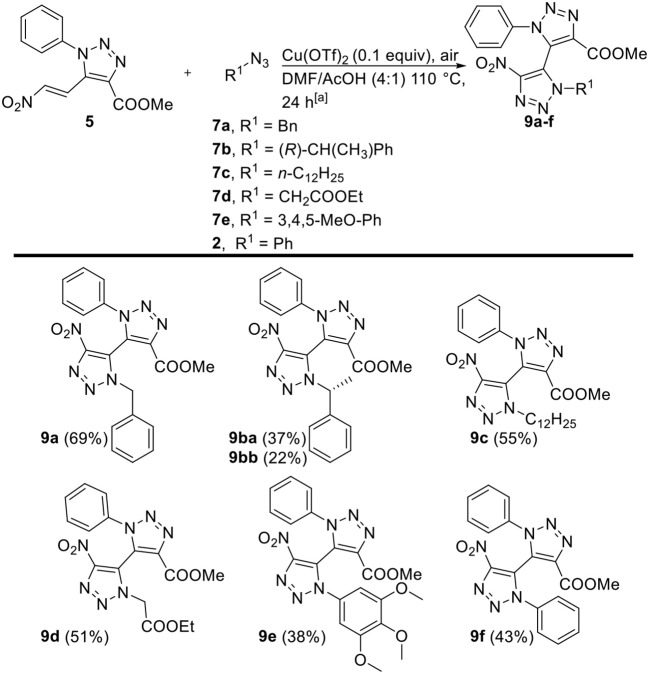
Scope of copper-catalyzed oxidative [3+2]-cycloaddition toward tetra-*ortho*-substituted 5,5′-bi-1,2,3-triazoles **9a–f**, variations with respect to 5-nitrovinyl-1,2,3-triazole **5**, and organic azides **2**, **7a–e**. ^[a]^ Reaction conditions: **5** (1 equiv), organic azides **2**, **7a**–**e** (1.5 equiv), Cu(OTf)_2_ (0.1 equiv), air, DMF/AcOH (2.5 mL, 4:1), 110°C.

The introduction of the nitro moiety on the 4-position of 1,2,3-triazoles is highly interesting in view of further transformations, yet tedious to accomplish with the three-component reaction since this would require the use of the hazardous dinitromethane at elevated temperatures. Hence, the copper-catalyzed oxidative [3+2]-cycloaddition reaction with 5-nitrovinyl-appended 1,2,3-triazole **5** serves as a complementairy pathway toward highly interesting 4-nitro substituted non-symmetrical tetra-*ortho*-substituted 5,5'-bi-1,2,3-triazoles. 5-Nitrovinyl-appended 1,2,3-triazole **5** was subjected to the oxidative [3+2]-cycloaddition conditions with different alkyl and aryl azides **2**, **7a**-**e** (Figure 2). Derivatives **9a**-**d**, obtained from alkyl azides **7a**-**d**, were obtained in moderate to good yields (51–70%). Again, the use of (*R*)-**7b** yielded a diastereomeric mixture of 1.77:1, which were successfully separated *via* silica gel chromatography and furnished both isolated diastereomers **9ba** and **9bb**. The use of aryl azides **2** and **7e** furnished derivatives **9e** and **9f**, whilst again 4-nitrophenyl azide did not furnish any desired product. In general, *via* this second pathway solely nitro derivatives can be obtained, and the crude reaction mixtures are cleaner than the ones obtained from the three-componentreaction with only the HNO_2_-eliminated derivatives as minor side products.

**Scheme 2:**
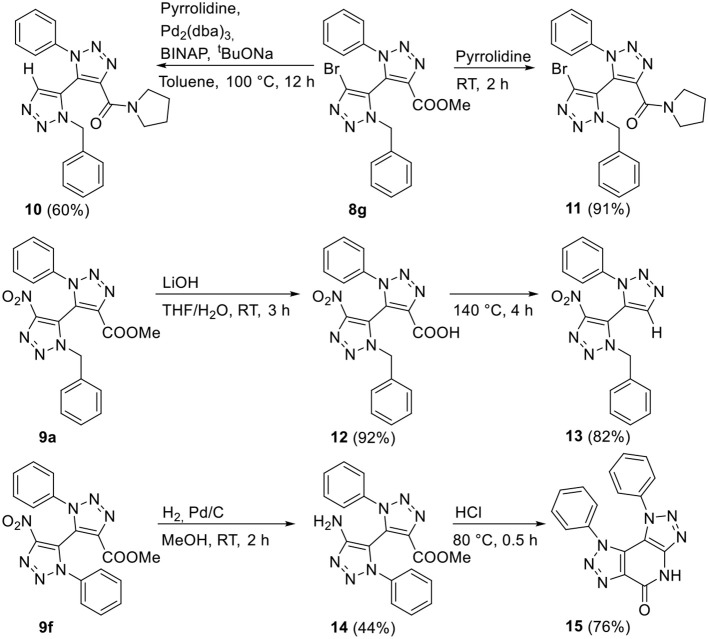
Postfunctionalization reactions performed with bi-1,2,3-triazoles **8g**, **9a**, and **9f**.

Several of the obtained bi-1,2,3-triazoles now bear interesting functionalities that could serve as valuable starting point for further derivatization (Scheme 2). Bromo-derivative **8g** was subjected to Buchwald-Hartwig amination conditions with pyrrolidine, yet surprisingly the reaction furnished hydrodehalogenated amide **10** in 60% yield. The reactivity of the ester moiety over the bromide of **8g** was further exemplified in a substitution reaction with pyrrolidine, in which amide **11** constituting the bromide formed in excellent yields upon reaction at room temperature. Next, hydrolysis of the methyl ester moiety of **9a** formed the highly versatile, yet possibly labile, carboxylic acid **12** in 92% yield. Hence, the carboxylic acid was in a next step subjected to heating in order to investigate its thermal stability. Carboxylic acid **12** displayed reasonable stability over the course of several weeks at room temperature, but at 140°C decarboxylation of **12** nicely furnished **13** in good yields (82%). Finally, the reduction of nitro derivative **9f** was investigated. Under standard hydrogenation conditions using Pd/C, the versatile free 4-amino-appended derivative **14** was obtained in 44% yield, without the observation of ring-closed amide **15**. Further cyclization toward bis-1,2,3-triazolo-fused 2-pyridone **15** was achieved under acidic conditions at 80°C, in which **15** was obtained in 76% yield.

**Figure 3:**
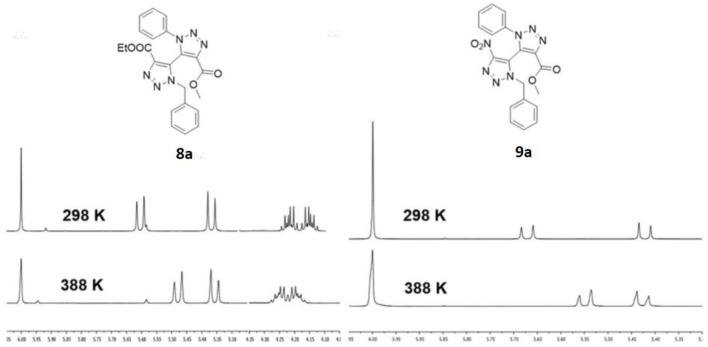
Variable temperature NMR studies for tetra-*ortho*-substituted **8a** and **9a**.

The newly prepared tetra-*ortho*-substituted 5,5'-bi-1,2,3-triazoles are fascinating structures in the viewpoint of obtaining novel 1,2,3-triazole-containing axially chiral derivatives. The presence of diastereotopic splitting displayed in the ^1^H NMR spectra of the synthesized products strongly indicates this existence of conformationally stable atropisomers (Figure 3). Hence, VT-NMR studies were conducted in tetrachloroethane-*d*_4_ for both **8a** and **9a** to have an estimate on the energy barrier to rotation for these novel bi-1,2,3-triazoles. At 25°C, both compounds show well-separated signals of the diastereotopic benzylic protons at 5.59 (*J* = 15.0 Hz) and 5.34 ppm (*J* = 15.0 Hz) for compound **8a**, and at 5.67 (*J* = 14.9 Hz) and 5.42 ppm (*J* = 14.9 Hz) for compound **9a**, both as clear AX spin systems. Moreover, compound **8a** also displayed split signals of the diastereotopic methylene protons of the ester group at 4.18 (*J* = 10.9, 7.1 Hz) and 4.11 ppm (*J* = 10.9, 7.1 Hz). Subsequently, from the high temperature NMR spectra at 115°C, it can be observed, based on the chemical shifts, that the diastereotopic signals of both compounds **8a** and **9a** move closer to each other when the temperature rises, i.e., benzylic signals of **8a** are positioned at 5.48 (*J* = 15.0 Hz) and 5.36 ppm (*J* = 15.0 Hz) at 115°C, the signals of the methylene protons of the ester are positioned at 4.25 (*J* = 10.8, 7.2 Hz) and 4.18 ppm (*J* = 10.8, 7.2 Hz) at 115°C, and for **9a** the benzylic signals are positioned at 5.55 (*J* = 14.9 Hz) and 5.43 ppm (*J* = 15.0 Hz) at 115°C (Figure 3). Unfortunately, or fortunately from the perspective of forming conformationally stable atropisomers, no coalescence was observed for the diastereotopic signals when the maximum temperature of 115°C (388 K) was reached. Therefore, the corresponding rotational energy barriers ΔG of these atropisomeric compounds **8a** and **9a** could not be accurately calculated. Nevertheless, a minimal energy could be tentatively calculated by assuming coalescence at 388 K. Hence, the rotational energy barrier at this temperature for both **8a** and **9a** is at least 79.5 kJ/mol. In addition, the isolation of both diastereomers *via* silica gel chromatography was possible for **8ba** and **8bb**, and **9ba** and **9bb**. Regrettably, the sets of diastereomers showcased a rather limited rotational stability as they isomerized at room temperature over the course of a couple of days. In future work, the steric hindrance of the *ortho*-substituents should be increased to create more stable axially chiral compounds. Nevertheless, this is an encouraging result from the perspective of preparing atropisomeric unsymmetrically substituted bi-1,2,3-triazoles.

## Conclusion

We report herein the synthesis toward unsymmetrically substituted 5,5'-bi-1,2,3-triazoles from 5-formyl-1,2,3-triazoles directly or indirectly *via* two distinct methodologies, i.e., a three-component reaction from 5-formyl-1,2,3-triazole, nitroalkanes, and organic azides yielding variously substituted 5,5'-bi-1,2,3-triazoles, and the copper-catalyzed oxidative [3+2]-cycloaddition reaction from 5-nitrovinyl-appended 1,2,3-triazole and organic azides resulting in 4-nitro-functionalized bi-1,2,3-triazoles. Both reactions displayed a versatile scope toward various alkyl and aryl azides, and *via* the use of both aforementioned procedures various attractive functional groups can be incorporated. Postfunctionalizations further emphasized their peculiar and interesting chemistries for future applications. Rotational stability tests showed promising characteristics as atropisomers, although the rotational barrier was still rather limited and could be further increased by implementing more sterically hindered *ortho*-substituents.

## Data Availability Statement

All datasets generated for this study are included in the article/Supplementary Material.

## Author Contributions

RV and TH carried out the experiments, analyzed the data, and wrote the manuscript. MV carried out some of the experiments and analyzed the data. WD directed the project and corrected the manuscript.

### Conflict of Interest

The authors declare that the research was conducted in the absence of any commercial or financial relationships that could be construed as a potential conflict of interest.
