# Supplementary material for: 5-Formyltriazoles as Valuable Starting Materials for Unsymmetrically Substituted Bi-1,2,3-Triazoles
Source: Front Chem. 2020 Apr 15;8:271. doi: 10.3389/fchem.2020.00271 (PMC7174647; doi:10.3389/fchem.2020.00271)
Supplement: Supplementary file 1 [file Table_1.DOCX]

*Supporting information for*

**5-Formyltriazoles as Valuable Starting Materials for Unsymmetrically Substituted Bi-1,2,3-triazoles**

Robby Vroemans^‡^, Tomas Horsten^‡^, Maarten Van Espen, and Wim Dehaen^*^

Molecular Design and Synthesis, Department of Chemistry, KU Leuven, Celestijnenlaan 200F, 3001 Leuven, Belgium.

^‡^ Both authors contributed equally to this work.

E-mail: Wim Dehaen - wim.dehaen@kuleuven.be

* Corresponding author

**Experimental part**

Chemicals received from commercial sources (Sigma Aldrich, Acros Organics, J&K Scientific, Alfa Aesar, Fluorochem or TCI Chemicals) were used without further purification. Organic azides **2,** **7a-e** (Campbell-Verduyn et al., 2009; Hu et al., 2008; Maddani et al., 2010; Titz et al., 2006) and Morph/TsOH (Thomas et al., 2014) used as starting materials in the reactions, were prepared according to known literature procedures. Dry reaction solvents were purchased from commercial sources. Thin-layer chromatography (TLC) was performed on silica gel 0.20 mm 60 with fluorescent indicator UV254 (pre-coated aluminum sheets) from Merck. For column chromatography 60-200 mesh silica gel 60 (Acros) was used as stationary phase. NMR spectra were acquired on commercial instruments (Bruker Avance 300 MHz, Bruker AMX 400 MHz or Bruker Avance II^+^ 600 MHz) and chemical shifts (δ) are reported in parts per million (ppm) referenced to tetramethylsilane (^1^H), or the internal (NMR) solvent signal (^13^C). High resolution mass spectra were acquired on a quadrupole orthogonal acceleration time-of-flight mass spectrometer (Synapt G2 HDMS, Waters, Milford, MA, USA). Samples were infused at 3µL/min and spectra were obtained in positive mode with a resolution of 15 000 (FWHM) using leucine enkephalin as lock mass. Melting points (uncorrected) were determined using a Reichert Thermovar apparatus.

# Methyl 5-(methoxymethyl)-1-phenyl-1*H*-1,2,3-triazole-4-carboxylate (3)

To a 500 mL round-bottom flask equipped with a magnetic stirring bar, methyl 4-methoxy acetoacetate **1** (56.45 g, 0.386 mol, 1 equiv) and phenyl azide **2** (55.2 g, 1.2 equiv) were dissolved in DMSO (200 mL). DBU (5.81 g, 0.1 equiv) was added dropwise and the mixture was stirred for three hours at room temperature. The solution was poured out into ice water, and the crude product was filtered over a Büchner funnel and washed with water. The crude product was crystallized from methanol, filtered, and dried in a vacuum oven at 50 °C overnight to obtain compound **3** in 81 % yield (78 g).

White crystals – Mp: 116 – 119 °C. ^1^H NMR (300MHz, CDCl_3_): *δ* (ppm) 7.73 – 7.63 (m, 2H), 7.64 – 7.49 (m, 3H), 4.76 (s, 2H), 4.02 (s, 3H), 3.42 (s, 3H). ^13^C NMR (75 MHz, CDCl_3_) *δ* (ppm) 161.94, 138.28, 137.75, 135.90, 130.26, 129.66, 125.23, 60.84, 58.58, 52.26. Exact mass (HRMS, ESI) calculated for C_12_H_13_N_3_O_3_ (M+H)^+^: 248.1030, found 248.1031.

# Methyl 5-formyl-1-phenyl-1*H*-1,2,3-triazole-4-carboxylate (4)

To a 1 L two-necked round-bottom flask equipped with a magnetic stirring bar and a reflux condenser, **3** (9.89 g, 40 mmol, 1 equiv) was refluxed in 500 mL chloroform while irradiated with a 500 W light bulb. Br_2_ (7.03 g, 44 mmol, 1.1 equiv) in 50 mL CHCl_3_ was added dropwise over two hours. The reaction was refluxed for an additional two hours. The mixture was cooled to room temperature, washed with water, saturated NaHCO_3_ and brine. The organic layer was dried over MgSO_4_, filtered, and concentrated under reduced pressure. The crude product was crystallized from diethyl ether to obtain compound **4** in 65 % yield (6.01 g).

White crystals – Mp: 96 – 100 °C. ^1^H NMR (400 MHz, CDCl_3_) *δ* (ppm) 10.55 (s, 1H), 7.62 – 7.51 (m, 3H), 7.48 – 7.43 (m, 2H), 4.09 (s, 3H). ^13^C NMR (101 MHz, CDCl_3_) *δ* (ppm) 179.82, 160.80, 142.45, 135.88, 134.88, 130.86, 129.35, 125.74, 53.19. Exact mass (HRMS, ESI) calculated for C_11_H_9_N_3_O_3_ (M+H)^+^: 232.0717, found 232.0718.

**Methyl 5-(2-nitrovinyl)-1-phenyl-1*H*-1,2,3-triazole-4-carboxylate (5)**

To an oven-dried screw-capped reaction tube equipped with a magnetic stirring bar, **4** (2.31 g, 10 mmol, 1 equiv), nitromethane (0.732 g, 12 mmol, 1.2 equiv) were dissolved in 10 mL dry DCM and cooled to 0 °C. 1,1,3,3-Tetramethylguanidine (TMG) (0.058 g, 0.5 mmol, 0.05 equiv) was added and the reaction was stirred for 5 minutes at 0 °C followed by 30 minutes at room temperature until a white precipitate is formed. Next, sodium acetate (0.820 g, 10 mmol, 1 equiv) and acetic anhydride (5.10 g, 50 mmol, 5 equiv) were added and the reaction was stirred at 50 °C for eight hours. The reaction mixture was diluted with DCM and washed with water, saturated NaHCO_3_, and brine. The organic layer was dried over MgSO_4_, filtered, and concentrated under reduced pressure. The crude product was crystallized from methanol to obtain compound **5** in 79 % yield (2.17 g).

Yellow crystals – Mp: 218 – 220 °C. ^1^H NMR (300 MHz, CDCl_3_): *δ* (ppm) 8.24 (d, *J* = 13.7 Hz, 1H), 7.93 (d, *J* = 13.7 Hz, 1H), 7.77 – 7.59 (m, 3H), 7.53 – 7.39 (m, 2H), 4.07 (s, 3H). ^13^C NMR (75 MHz, CDCl3): *δ* (ppm) 161.53, 144.48, 138.83, 134.75, 132.01, 131.66, 130.52, 126.06, 120.32, 53.10. Exact mass (HRMS, ESI) calculated for C_12_H_10_N_4_O_4_ (M+H)^+^: 275.0775, found 275.0781.

**General procedure A: Synthesis of bi-1,2,3-triazoles 8a-i**

To an oven-dried screw-capped reaction tube equipped with a magnetic stirring bar, **4** (100 mg, 0.43 mmol, 1 equiv), the corresponding nitroalkane **6a-d** (0.56 mmol, 1.3 equiv), organic azide **2, 7a-e** (0.86 mmol, 2 equiv), BHT (19 mg, 0.09 mmol, 0.2 equiv), Morph/TsOH (21 mg, 0.09 mmol, 0.2 equiv), 4 Å molecular sieves (50 mg), and 0.2 mL dry toluene were added. The mixture was stirred at 110 °C under inert atmosphere. Reaction was monitored by TLC. After completion of the reaction, the mixture was cooled to room temperature. The crude reaction mixture was purified *via* flash chomatography over silica with petroleum ether / ethyl acetate as eluent affording the pure compounds **8a-i**.

**Methyl 3'-benzyl-3-phenyl-5'-(propionyloxy)-3*H*,3'*H*-[4,4'-bi(1,2,3-triazole)]-5-carboxylate (8a)**

Prepared according to general procedure A: Reaction time 20 h. Ethyl nitroacetate **6a** (75 mg, 0.56 mmol), benzyl azide **7a** (100 mg, 0.75 mmol). Compound **8a** was obtained in 87 % yield (188 mg).

White crystals – Mp: 118 – 120 °C. ^1^H NMR (400 MHz, CDCl_3_) *δ* (ppm) 7.36 – 7.21 (m, 4H), 7.21 – 7.15 (m, 2H), 7.03 – 6.96 (m, 2H), 6.61 – 6.55 (m, 2H), 5.67 (d, *J* = 14.9 Hz, 1H), 5.37 (d, *J* = 14.9 Hz, 1H), 4.24 – 4.07 (m, 2H), 3.91 (s, 3H), 1.16 (t, *J* = 7.1 Hz, 3H). ^1^H NMR (600 MHz, Cl_2_CDCDCl_2_) *δ* (ppm) 7.37 (t, *J* = 7.5 Hz, 1H), 7.30 (d, *J* = 2.0 Hz, 1H), 7.26 – 7.20 (m, 4H), 6.97 (d, *J* = 7.3 Hz, 2H), 6.63 – 6.58 (m, 2H), 5.59 (d, *J* = 15.0 Hz, 1H), 5.34 (d, *J* = 15.0 Hz, 1H), 4.18 (dq, *J* = 10.9, 7.1 Hz, 1H), 4.11 (dq, *J* = 10.9, 7.1 Hz, 1H), 3.87 (s, 3H), 1.13 (t, *J* = 7.1 Hz, 3H). ^13^C NMR (101 MHz, CDCl_3_) *δ* (ppm) 160.56, 159.49, 140.11, 138.96, 134.92, 132.90, 130.29, 129.53, 129.44, 129.42, 128.59, 127.40, 126.98, 123.90, 61.71, 54.13, 52.84, 14.10. Exact mass (HRMS, ESI) calculated for C_22_H_20_N_6_O_4_ (M+H)^+^: 433.1618, found 433.1614.

**5-Ethyl 5'-methyl (*R*)-3'-phenyl-3-(1-phenylethyl)-3*H*,3'*H*-[4,4'-bi(1,2,3-triazole)]-5,5'-dicarboxylate (8b)**

Prepared according to general procedure A: Reaction time 20 h. Ethyl nitroacetate **6a** (75 mg, 0.56 mmol), (*R*)-(1-azidoethyl)benzene **7b** (147 mg, 1 mmol, 2 equiv). A mixture of diastereomers was obtained in 56 % yield (108 mg). The ratio of both diastereomers was estimated from the crude ^1^H NMR spectrum and was 2.25:1 (**8ba**/**8bb**). Diastereomers were separated *via* flash chromatography over silica with petroleum ether / MTBE.

Diastereomer **8ba**: White semi-solid. ^1^H NMR (300 MHz, CDCl_3_) *δ* (ppm) 7.26 (d, *J* = 6.4 Hz, 4H), 7.12 – 7.00 (m, 4H), 6.40 (d, *J* = 7.9 Hz, 2H), 5.53 (q, *J* = 6.9 Hz, 1H), 4.19 – 4.04 (m, 2H), 3.99 (s, 3H), 2.15 (d, *J* = 7.0 Hz, 3H), 1.14 (t, *J* = 7.1 Hz, 3H). ^13^C NMR (75 MHz, CDCl_3_) *δ* (ppm) 160.77, 159.56, 139.28, 139.17, 138.64, 134.84, 130.24, 130.07, 129.47, 129.16, 127.61, 127.06, 126.74, 124.05, 61.59, 61.27, 52.94, 21.80, 14.08. Exact mass (HRMS, ESI) calculated for C_23_H_22_N_6_O_4_ (M+H)^+^: 447.1775, found 447.1767.

Diastereomer **8bb**: White semi-solid. ^1^H NMR (300 MHz, CDCl_3_) *δ* (ppm) 7.55 – 7.45 (m, 3H), 7.41 – 7.33 (m, 2H), 7.26 – 7.18 (m, 3H), 7.03 – 6.93 (m, 2H), 4.83 (q, *J* = 7.0 Hz, 1H), 4.39 – 4.22 (m, 2H), 3.46 (s, 3H), 1.76 (d, *J* = 7.0 Hz, 3H), 1.22 (d, *J* = 7.1 Hz, 3H). ^13^C NMR (75 MHz, CDCl_3_) *δ* (ppm) 159.93, 159.36, 140.85, 140.02, 138.54, 135.12, 130.82, 130.24, 129.07, 128.94, 127.19, 127.16, 126.14, 123.86, 61.77, 61.17, 52.13, 21.70, 14.15. Exact mass (HRMS, ESI) calculated for C_23_H_22_N_6_O_4_ (M+H)^+^: 447.1775, found 447.1764.

**Methyl 3'-dodecyl-3-phenyl-5'-(propionyloxy)-3*H*,3'*H*-[4,4'-bi(1,2,3-triazole)]-5-carboxylate (8c)**

Prepared according to general procedure A: Reaction time 20 h. Ethyl nitroacetate **6a** (75 mg, 0.56 mmol), dodecyl azide **7c** (183 mg, 0.75 mmol). Compound **8c** was obtained in 63 % yield (140 mg).

Yellow oil. ^1^H NMR (300 MHz, CDCl_3_) *δ* (ppm) 7.51 – 7.40 (m, 3H), 7.30 (d, *J* = 6.9 Hz, 2H), 4.32 – 4.17 (m, 2H), 4.14 – 3.98 (m, 2H), 3.90 (s, 3H), 1.77 (dd, *J* = 13.8, 6.8 Hz, 1H), 1.66 (dd, *J* = 13.7, 6.7 Hz, 1H), 1.30 – 1.14 (m, 21H), 0.88 (t, *J* = 6.5 Hz, 3H). ^13^C NMR (75 MHz, CDCl_3_) *δ* (ppm) 160.32, 159.82, 139.89, 139.72, 135.22, 130.69, 129.99, 127.44, 127.05, 123.97, 61.76, 52.80, 49.84, 32.02, 29.69, 29.56, 29.44, 29.43, 29.34, 28.99, 26.53, 22.80, 14.24, 14.16. Exact mass (HRMS, ESI) calculated for C_27_H_38_N_6_O_4_ (M+H)^+^: 511.3027, found 511.3025.

**Methyl 3'-(2-ethoxy-2-oxoethyl)-3-phenyl-5'-(propionyloxy)-3*H*,3'*H*-[4,4'-bi(1,2,3-triazole)]-5-carboxylate (8d)**

Prepared according to general procedure A: Reaction time 20 h. Ethyl nitroacetate **6a** (75 mg, 0.56 mmol), ethyl 2-azidoacetate **7d** (112 mg, 0.75 mmol). Compound **8d** was obtained in 74 % yield (137 mg).

Off-white solid – Mp: 156 – 159 °C. ^1^H NMR (300 MHz, CDCl_3_) *δ* (ppm) 7.54 (d, *J* = 6.6 Hz, 2H), 7.42 (d, *J* = 6.2 Hz, 3H), 5.46 (d, *J* = 17.8 Hz, 1H), 5.22 (d, *J* = 17.8 Hz, 1H), 4.25 – 3.99 (m, 4H), 3.94 (s, 3H), 1.24 (t, *J* = 7.1 Hz, 3H), 1.11 (t, *J* = 7.0 Hz, 3H). ^13^C NMR (75 MHz, CDCl_3_) *δ* (ppm) 165.89, 160.74, 159.02, 138.96, 138.55, 135.41, 130.10, 129.38, 128.27, 126.61, 124.04, 62.82, 61.57, 52.82, 50.61, 13.93, 13.90. Exact mass (HRMS, ESI) calculated for C_19_H_20_N_6_O_6_ (M+H)^+^: 429.1517, found 429.1511.

**Methyl 3-phenyl-5'-(propionyloxy)-3'-(3,4,5-trimethoxyphenyl)-3*H*,3'*H*-[4,4'-bi(1,2,3-triazole)]-5-carboxylate (8e)**

Prepared according to general procedure A: Reaction time 48 h. Ethyl nitroacetate **6a** (75 mg, 0.56 mmol), 3,4,5-trimethoxyphenyl azide **7e** (142 mg, 0.75 mmol). Compound **8e** was obtained in 21 % yield (45 mg).

Brown solid – Mp: 140 – 144 °C. ^1^H NMR (300 MHz, CDCl_3_) *δ* (ppm) 7.43 (t, *J* = 7.4 Hz, 1H), 7.33 (t, *J* = 7.6 Hz, 2H), 6.97 (d, *J* = 7.7 Hz, 2H), 6.10 (s, 2H), 4.37 (m, 2H), 3.94 (s, 3H), 3.84 (s, 3H), 3.62 (s, 6H), 1.32 (t, *J* = 7.1 Hz, 3H). ^13^C NMR (75 MHz, CDCl_3_) *δ* (ppm) 160.47, 159.78, 153.73, 140.67, 140.40, 139.47, 134.95, 130.33, 130.09, 129.77, 127.67, 127.09, 124.00, 101.56, 62.06, 61.13, 56.32, 52.84, 14.18. Exact mass (HRMS, ESI) calculated for C_24_H_24_N_6_O_7_ (M+H)^+^: 509.1779, found 509.1778.

**Methyl 3,3'-diphenyl-5'-(propionyloxy)-3*H*,3'*H*-[4,4'-bi(1,2,3-triazole)]-5-carboxylate (8f)**

Prepared according to general procedure A: Reaction time 48 h. Ethyl nitroacetate **6a** (75 mg, 0.56 mmol), phenyl azide **2** (142 mg, 0.75 mmol). Compound **8f** was obtained in 36 % yield (65 mg).

Orange oil. ^1^H NMR (300 MHz, CDCl_3_) *δ* (ppm) 7.63 – 7.58 (m, 1H), 7.42 (t, *J* = 7.4 Hz, 2H), 7.35 – 7.26 (m, 4H), 6.93 (d, *J* = 7.9 Hz, 3H), 4.46 – 4.28 (m, 2H), 3.91 (s, 3H), 1.31 (t, *J* = 7.2 Hz, 3H). ^13^C NMR (75 MHz, CDCl_3_) *δ* (ppm) 160.28, 159.79, 140.63, 140.33, 134.90, 134.87, 130.43, 129.83, 129.76, 127.44, 127.10, 127.07, 123.98, 123.95, 62.01, 52.76, 14.17. Exact mass (HRMS, ESI) calculated for C_21_H_18_N_6_O_4_ (M+H)^+^: 419.1462, found 419.1458.

**Methyl 3'-benzyl-5'-bromo-3-phenyl-3*H*,3'*H*-[4,4'-bi(1,2,3-triazole)]-5-carboxylate (8g)**

To an oven-dried screw-capped reaction tube equipped with a magnetic stirring bar, **4** (116 mg, 0.5 mmol, 1 equiv) and bromonitromethane **6b** (84 mg, 0.6 mmol, 1.2 equiv) were dissolved in 0.4 mL dry DCM, and cooled to 0 °C. TMG (5,76 mg, 0.05, 0.1 equiv) was added and the reaction was stirred for five minutes at 0 °C, followed by 30 minutes at room temperature. Next, sodium acetate (42 mg, 0.5 mmol, 1 equiv) and acetic anhydride (510 mg, 5 mmol, 10 equiv) were added and the reaction was stirred at 50 °C for eight hours. The crude reaction mixture was diluted with DCM and extracted with water and brine. The organic layer was dried over MgSO_4_, filtered, and dried under reduced pressure to obtain the crude bromonitroalkene as a yellow semisolid. To a 10 mL round-bottom flask equipped with a magnetic stirring bar and the crude mixture was added benzyl azide **7a** (100 mg, 0.75 mmol, 1.5 equiv), BHT (11 mg, 0.05 mmol, 0.1 equiv), TsOH·H_2_O (10 mg, 0.05 mmol, 0.1 equiv), and 1 mL dry toluene. The reaction mixture was stirred at 110 °C for 60 hours. The crude product was purified *via* flash chromatography over silica with petroleum ether / ethyl acetate as eluent affording compound **8g** in 64 % yield (140 mg).

Light brown solid – Mp: 118 – 121 °C. ^1^H NMR (300 MHz, CDCl_3_) *δ* (ppm) 7.44 – 7.35 (m, 1H), 7.32 – 7.17 (m, 5H), 7.03 – 6.92 (m, 2H), 6.71 – 6.59 (m, 2H), 5.57 (d, *J* = 14.8 Hz, 1H), 5.39 (d, *J* = 14.8 Hz, 1H), 3.94 (s, 3H). ^13^C NMR (75 MHz, CDCl_3_) *δ* (ppm) 160.31, 139.62, 134.75, 132.90, 130.46, 129.68, 129.50, 129.36, 128.47, 126.50, 124.62, 123.80, 122.82, 54.87, 52.88. Exact mass (HRMS, ESI) calculated for C_19_H_15_BrN_6_O_2_ (M+H)^+^: 439.0513, found 439.0512.

**Methyl 5'-benzoyl-3'-benzyl-3-phenyl-3*H*,3'*H*-[4,4'-bi(1,2,3-triazole)]-5-carboxylate (8h)**

Prepared according to general procedure A: Reaction time 20 h. Benzoylnitromethane **6c** (93 mg, 0.56 mmol), benzyl azide **7a** (142 mg, 0.75 mmol). Compound **8h** was obtained in 54 % yield (108 mg).

Orange oil. ^1^H NMR (300 MHz, CDCl_3_) *δ* (ppm) 8.15 (d, *J* = 7.4 Hz, 2H), 7.56 (dd, *J* = 10.3, 4.1 Hz, 2H), 7.46 – 7.40 (m, 3H), 7.25 (s, *J* = 7.2 Hz, 2H), 7.12 (t, *J* = 7.8 Hz, 2H), 7.02 (d, *J* = 6.3 Hz, 2H), 6.69 (d, *J* = 7.9 Hz, 2H), 5.61 (d, *J* = 14.9 Hz, 1H), 5.43 (d, *J* = 14.9 Hz, 1H), 3.87 (s, 3H). ^13^C NMR (75 MHz, CDCl_3_) *δ* (ppm) 185.19, 160.59, 147.02, 138.54, 135.99, 134.93, 133.63, 132.90, 130.52, 130.19, 129.44, 129.37, 129.31, 128.52, 128.39, 128.27, 127.67, 124.14, 53.91, 52.69. Exact mass (HRMS, ESI) calculated for C_26_H_20_N_6_O_3_ (M+H)^+^: 465.1670, found 465.1664.

**Methyl 3'-benzyl-3-phenyl-5'-(phenylsulfonyl)-3*H*,3'*H*-[4,4'-bi(1,2,3-triazole)]-5-carboxylate (8i)**

Prepared according to general procedure A: Reaction time 20 h. Phenylsulfonylnitromethane **6d** (113 mg, 0.56 mmol), benzyl azide **7a** (142 mg, 0.75 mmol). Compound **8i** was obtained in 54 % yield (117 mg).

Off-white solid – Mp: 137 – 143 °C. ^1^H NMR (300 MHz, CDCl_3_) *δ* (ppm) 7.64 (d, *J* = 7.6 Hz, 2H), 7.58 (t, *J* = 7.4 Hz, 1H), 7.46 – 7.36 (m, 3H), 7.28 – 7.18 (m, 5H), 6.94 (d, *J* = 7.1 Hz, 2H), 6.87 (d, *J* = 7.9 Hz, 2H), 5.46 (d, *J* = 14.9 Hz, 1H), 5.30 (d, *J* = 14.9 Hz, 1H), 3.66 (s, 3H). ^13^C NMR (75 MHz, CDCl_3_) *δ* (ppm) 159.89, 148.27, 139.65, 139.40, 134.89, 134.21, 131.98, 130.39, 129.70, 129.59, 129.37, 129.28, 128.49, 128.07, 125.63, 125.29, 123.98, 54.31, 52.54. Exact mass (HRMS, ESI) calculated for C_25_H_20_N_6_O_4_S (M+H)^+^: 501.1339, found 501.1335.

**General procedure B: Synthesis of 4-nitro-bi-1,2,3-triazoles 9a-f**

To an oven-dried flask equipped with a magnetic stirring bar were added **5** (137 mg, 0.5 mmol, 1 equiv), organic azide **2, 7a-e** (0.75 mmol, 1.5 equiv), and Cu(OTf)_2_ (18 mg, 0.1 mmol, 0.1 equiv). The mixture was dissolved in DMF/AcOH (2.5 mL, v/v, 4:1), and subsequently stirred at 110 °C under air atmosphere (1 atm). The reaction was monitored by TLC. After completion of the reaction, the mixture was cooled to room temperature and diluted with EtOAc. The organic phase was washed with water, saturated NaHCO_3_, and brine. The organic layer was dried over MgSO_4_, filtered, and concentrated under reduced pressure. The crude product was purified *via* flash chromatography over silica with petroleum ether / ethyl acetate as eluent affording the pure compounds **9a-f**.

**Methyl 3'-benzyl-5'-nitro-3-phenyl-3*H*,3'*H*-[4,4'-bi(1,2,3-triazole)]-5-carboxylate (9a)**

Prepared according to general procedure B: Reaction time 20 h. Benzyl azide **7a** (100 mg, 0.75 mmol). Compound **9a** was obtained in 69 % yield (139 mg).

White solid – Mp: 185 – 190 °C. ^1^H NMR (400 MHz, CDCl_3_) *δ* (ppm) 7.41 – 7.27 (m, 4H), 7.24 – 7.18 (m, 2H), 7.06 – 7.01 (m, 2H), 6.60 – 6.48 (m, 2H), 5.71 (d, *J* = 14.9 Hz, 1H), 5.43 (d, *J* = 14.9 Hz, 1H), 3.96 (s, 3H). ^1^H NMR (600 MHz, Cl_2_CDCDCl_2_) *δ* (ppm) 7.43 – 7.40 (m, 1H), 7.38 – 7.34 (m, 1H), 7.31 (d, *J* = 7.8 Hz, 2H), 7.27 – 7.24 (m, 2H), 7.04 – 6.97 (m, 2H), 6.55 (dd, *J* = 8.5, 1.0 Hz, 2H), 5.67 (d, *J* = 14.9 Hz, 1H), 5.42 (d, *J* = 14.9 Hz, 1H), 3.94 (s, 3H). ^13^C NMR (101 MHz, CDCl_3_) *δ* (ppm) 160.42, 139.41, 134.49, 131.89, 130.92, 130.05, 129.83, 129.70, 128.74, 125.35, 123.77, 122.19, 55.41, 53.14. Exact mass (HRMS, ESI) calculated for C_19_H_15_N_7_O_4_ (M+H)^+^: 406.1258, found 406.1254.

**Methyl 5'-nitro-3-phenyl-3'-((*R*)-1-phenylethyl)-3*H*,3'*H*-[4,4'-bi(1,2,3-triazole)]-5-carboxylate (9b)**

Prepared according to general procedure B: Reaction time 25 h. (*R*)-(1-azidoethyl)benzene **7b** (147 mg, 1 mmol, 2 equiv). A mixture of diastereomers was obtained in 59 % yield (124 mg). The ratio of both diastereomers was estimated from the crude ^1^H NMR spectrum taken after extraction and was 1.77:1 (**9ba**/**9bb**). Diastereomers were separated *via* flash chromatography over silica with petroleum ether / MTBE.

Diastereomer **9ba**: White solid – Mp: 49 – 54 °C. ^1^H NMR (400 MHz, DMSO-*d_6_*) *δ* (ppm) 7.46 – 7.40 (m, 1H), 7.33 – 7.28 (m, 3H), 7.27 – 7.21 (m, 2H), 7.15 – 7.10 (m, 2H), 6.67 – 6.61 (m, 2H), 5.97 (q, *J* = 6.8 Hz, 1H), 3.87 (s, 3H), 1.94 (d, *J* = 6.8 Hz, 3H). ^13^C NMR (101 MHz, DMSO) *δ* (ppm) 159.61, 139.63, 138.02, 133.99, 130.77, 129.80, 129.14, 129.00, 126.75, 124.39, 123.41, 122.31, 61.00, 52.76, 21.15. Exact mass (HRMS, ESI) calculated for C_20_H_17_N_7_O_4_ (M+H)^+^: 420.1415, found 420.1412.

Diastereomer **9bb**: White solid – Mp: 45 – 48 °C. ^1^H NMR (400 MHz, DMSO-*d_6_*) *δ* (ppm) 7.69 – 7.59 (m, 5H), 7.33 – 7.29 (m, 3H), 7.14 – 7.07 (m, 2H), 5.46 (q, *J* = 6.9 Hz, 1H), 3.43 (s, 3H), 1.54 (d, *J* = 6.9 Hz, 3H). ^13^C NMR (101 MHz, DMSO) *δ* (ppm) 158.92, 151.73, 139.50, 138.19, 134.11, 131.32, 130.43, 128.77, 128.68, 126.25, 124.90, 124.55, 122.33, 60.56, 52.22, 20.62. Exact mass (HRMS, ESI) calculated for C_20_H_17_N_7_O_4_ (M+H)^+^: 420.1415, found 420.1408.

**Methyl 3'-dodecyl-5'-nitro-3-phenyl-3*H*,3'*H*-[4,4'-bi(1,2,3-triazole)]-5-carboxylate (9c)**

Prepared according to general procedure B: Reaction time 20 h. Dodecyl azide **7c** (158 mg, 0.75 mmol). Compound **9c** was obtained in 55 % yield (133 mg).

White solid – Mp: 66 – 69 °C. ^1^H NMR (300 MHz, CDCl_3_) *δ* (ppm) 7.58 – 7.43 (m, 3H), 7.33 – 7.25 (m, 2H), 4.21 – 3.99 (m, 2H), 3.93 (s, 3H), 1.91 – 1.63 (m, 2H), 1.33 – 1.14 (m, 18H), 0.88 (t, *J* = 6.3 Hz, 3H). ^13^C NMR (75 MHz, CDCl_3_) *δ* (ppm) 160.11, 140.09, 134.77, 131.28, 130.35, 125.36, 123.96, 122.34, 53.08, 50.95, 32.00, 29.67, 29.53, 29.42, 29.37, 29.02, 28.93, 26.45, 22.79, 14.23. Exact mass (HRMS, ESI) calculated for C_24_H_33_N_7_O_4_ (M+H)^+^: 484.2666, found 484.2662.

**Methyl 3'-(2-ethoxy-2-oxoethyl)-5'-nitro-3-phenyl-3*H*,3'*H*-[4,4'-bi(1,2,3-triazole)]-5-carboxylate (9d)**

Prepared according to general procedure B: Reaction time 20 h. Ethyl 2-azidoacetate **7d** (97 mg, 0.75 mmol). Compound **9d** was obtained in 51 % yield (103 mg).

White solid – Mp: 81 – 85 °C. ^1^H NMR (400 MHz, CDCl_3_) *δ* (ppm) 7.60 – 7.53 (m, 2H), 7.53 – 7.40 (m, 3H), 5.51 (d, *J* = 17.8 Hz, 1H), 5.28 (d, *J* = 17.8 Hz, 1H), 4.33 – 4.12 (m, 2H), 3.98 (s, 3H), 1.27 (t, *J* = 7.2 Hz, 3H). ^13^C NMR (101 MHz, CDCl_3_) *δ* (ppm) 165.40, 160.86, 150.70, 139.16, 135.18, 130.94, 129.99, 124.81, 124.06, 123.72, 63.47, 53.28, 51.76, 14.06. Exact mass (HRMS, ESI) calculated for C_16_H_15_N_7_O_6_ (M+H)^+^: 402.1156, found 402.1155.

**Methyl 5'-nitro-3-phenyl-3'-(3,4,5-trimethoxyphenyl)-3*H*,3'*H*-[4,4'-bi(1,2,3-triazole)]-5-carboxylate (9e)**

Prepared according to general procedure B: Reaction time 20 h. 3,4,5-Trimethoxyphenyl azide **7e** (158 mg, 0.75 mmol). Compound **9e** was obtained in 38 % yield (91 mg).

White solid – Mp: 183 – 187 °C. ^1^H NMR (400 MHz, CDCl_3_) *δ* (ppm) 7.53 – 7.44 (m, 1H), 7.42 – 7.35 (m, 2H), 7.04 – 6.96 (m, 2H), 6.08 (s, 2H), 3.97 (s, 3H), 3.86 (s, 3H), 3.64 (s, 6H). ^13^C NMR (75 MHz, CDCl_3_) *δ* (ppm) 160.21, 153.83, 152.29, 140.60, 139.98, 134.50, 130.69, 130.03, 129.38, 125.49, 123.82, 122.32, 101.45, 61.09, 56.35, 53.12. Exact mass (HRMS, ESI) calculated for C_21_H_19_N_7_O_7_ (M+H)^+^: 482.1418, found 482.1411.

**Methyl 5'-nitro-3,3'-diphenyl-3*H*,3'*H*-[4,4'-bi(1,2,3-triazole)]-5-carboxylate (9f)**

Prepared according to general procedure A: Reaction time 40 h. Phenyl azide **2** (90 mg, 0.75 mmol). Compound **9f** was obtained in 43 % yield (84 mg).

White solid – Mp: 172 – 175 °C. ^1^H NMR (300 MHz, CDCl_3_) *δ* (ppm) 7.54 – 7.41 (m, 2H), 7.41 – 7.32 (m, 4H), 7.00 – 6.88 (m, 4H), 3.93 (s, 3H). ^13^C NMR (75 MHz, CDCl_3_) *δ* (ppm) 160.07, 140.65, 134.52, 134.41, 131.21, 130.90, 130.07, 130.03, 125.40, 123.99, 123.87, 122.45, 53.04. Exact mass (HRMS, ESI) calculated for C_18_H_13_N_7_O_4_ (M+H)^+^: 392.1101, found 392.1095.

**(3'-Benzyl-3-phenyl-3*H*,3'*H*-[4,4'-bi(1,2,3-triazol)]-5-yl)(pyrrolidin-1-yl)methanone (10)**

To an oven-dried screw-capped reaction tube equipped with a magnetic stirring bar, **8g** (150 mg, 0.34 mmol, 1 equiv), pyrrolidine (29 mg, 0.41 mmol, 1.2 equiv), Pd_2_(dba)_3_ (16 mg, 0.017 mmol, 0.05 equiv), BINAP (32 mg, 0.051 mmol, 0.15 equiv), and sodium *tert*-butoxide (49 mg, 0.51 mmol, 1.5 equiv) were dissolved in 0.6 mL toluene and stirred at 100 °C for 12 hours. The crude reaction mixture was purified *via* flash chromatography over silica with petroleum ether / ethyl acetate as eluent affording compound **10** in 60 % yield (82 mg).

White solid – Mp: 72 – 74 °C. ^1^H NMR (400 MHz, CDCl_3_) *δ* (ppm) 7.52 (s, 1H), 7.33 (t, *J* = 7.5 Hz, 1H), 7.24 – 7.16 (m, 5H), 7.01 – 6.94 (m, 2H), 6.57 – 6.49 (m, 2H), 5.74 (s, 2H), 4.06 (t, *J* = 6.7 Hz, 2H), 3.65 (t, *J* = 6.8 Hz, 2H), 2.07 – 1.99 (m, 2H), 1.99 – 1.91 (m, 2H). ^13^C NMR (101 MHz, CDCl_3_) *δ* (ppm) 158.84, 143.29, 136.48, 134.66, 134.37, 130.00, 129.24, 128.96, 128.67, 128.39, 127.15, 124.44, 123.54, 53.49, 48.99, 46.98, 26.52, 23.83. Exact mass (HRMS, ESI) calculated for C_22_H_21_N_7_O (M+H)^+^: 400.1880, found 400.1878.

**(3'-Benzyl-5'-bromo-3-phenyl-3*H*,3'*H*-[4,4'-bi(1,2,3-triazol)]-5-yl)(pyrrolidin-1-yl)methanone (11)**

To an oven-dried screw-capped reaction tube equipped with a magnetic stirring bar, **8g** (88 mg, 0.2 mmol, 1 equiv) and 0.5 mL pyrrolidine was stirred at room temperature for three hours. The reaction mixture was diluted with water and washed three times with DCM. The organic layer was dried over MgSO_4_, filtered, and concentrated under reduced pressure. The crude product was purified *via* flash chromatography over silica with DCM / ethyl acetate as eluent affording compound **11** in 91 % yield (87 mg).

White solid – Mp: 154 – 157 °C. ^1^H NMR (400 MHz, CDCl_3_) *δ* (ppm) 7.35 – 7.29 (m, 1H), 7.29 – 7.19 (m, 3H), 7.18 – 7.13 (m, 2H), 7.08 – 7.00 (m, 2H), 6.51 – 6.43 (m, 2H), 5.84 (d, *J* = 14.8 Hz, 1H), 5.77 (d, *J* = 14.8 Hz, 1H), 4.34 – 4.26 (m, 1H), 4.00 – 3.93 (m, 1H), 3.72 – 3.61 (m, 1H), 2.16 – 1.92 (m, 3H). ^13^C NMR (101 MHz, CDCl_3_) *δ* (ppm) 158.75, 144.12, 134.98, 133.73, 130.04, 129.35, 129.27, 129.22, 128.90, 125.50, 124.14, 123.96, 123.69, 55.01, 49.19, 47.18, 26.64, 23.98. Exact mass (HRMS, ESI) calculated for C_22_H_20_BrN_7_O (M+H)^+^: 478.0986, found 478.0980.

**3'-Benzyl-5'-nitro-3-phenyl-3*H*,3'*H*-[4,4'-bi(1,2,3-triazole)]-5-carboxylic acid (12)**

To a 10 mL round-bottom flask equipped with a magnetic stirring bar, **9a** (203 mg, 0.5 mmol, 1 equiv) and LiOH·H_2_O (63 mg, 1.5 mmol, 3 equiv) were dissolved in THF/H_2_O (3 mL, v/v, 1:1) and stirred at room temperature for three hours untill full conversion as monitored by TLC. The pH of the reaction mixture was adjusted to pH = 4 with 1 M HCl and extracted three times with EtOAc. The organic layer was dried over MgSO_4_, filtered, and concentrated under reduced pressure to obtain compound **12** in 92 % yield (180 mg).

White solid – Mp: 161 – 166 °C. ^1^H NMR (400 MHz, CDCl_3_) *δ* (ppm) 7.42 – 7.37 (m, 1H), 7.37 – 7.27 (m, 3H), 7.25 – 7.19 (m, 2H), 7.06 – 7.00 (m, 2H), 6.62 – 6.38 (m, 2H), 5.73 (d, *J* = 14.9 Hz, 1H), 5.44 (d, *J* = 14.9 Hz, 1H). ^13^C NMR (101 MHz, CDCl_3_) *δ* (ppm) 161.32, 138.90, 134.42, 131.86, 131.09, 130.14, 129.90, 129.79, 128.76, 125.77, 123.81, 121.85, 55.55. Exact mass (HRMS, ESI) calculated for C_18_H_13_N_7_O_4_ (M+H)^+^: 392.1102, found 392.1100.

**3-Benzyl-5-nitro-3'-phenyl-3*H*,3'*H*-4,4'-bi(1,2,3-triazole) (13)**

To a screw-capped reaction tube equipped with a magnetic stirring bar, **12** (78 mg, 0.2 mmol, 1 equiv) was heated at 140 °C for 4 hours. The reaction was cooled to room temperature, diluted with ethyl acetate, and washed with saturated NaHCO_3_ and brine. The organic layer was dried over MgSO_4_, filtered, and concentrated under reduced pressure to obtain compound **13** in 82 % yield (57 mg).

White solid – Mp: 142 – 145 °C. ^1^H NMR (400 MHz, CDCl_3_) *δ* (ppm) 7.90 (s, 1H), 7.42 – 7.22 (m, 6H), 7.07 – 6.96 (m, 2H), 6.76 – 6.69 (m, 2H), 5.53 (s, 2H). ^13^C NMR (75 MHz, CDCl_3_) *δ* (ppm) 136.05, 135.20, 132.10, 130.38, 129.93, 129.82, 129.69, 128.21, 123.57, 123.26, 121.25, 54.65.

**Methyl 5'-amino-3,3'-diphenyl-3*H*,3'*H*-[4,4'-bi(1,2,3-triazole)]-5-carboxylate (14)**

To a 50 mL round-bottom flask equipped with a magnetic stirring bar, **9f** (78 mg, 0,2 mmol, 1 equiv) was dissolved in 20 mL MeOH and flushed with N_2_. Pd/C 10 wt% (5 mg) was added under N_2_ atmosphere, and the reaction was stirred at room temperature under H_2_ atmosphere for two hours. The mixture was filtered over celite and MeOH was removed under reduced pressure. The crude product was purified *via* flash chromatography over silica with petroleum ether / ethyl acetate as eluent affording compound **14** in 44 % yield (32 mg).

White solid – Mp: 126 – 130 °C. ^1^H NMR (400 MHz, CDCl_3_) *δ* (ppm) 7.36 – 7.30 (m, 1H), 7.28 – 7.23 (m, 1H), 7.22 – 7.16 (m, 2H), 7.15 – 7.10 (m, 2H), 6.76 – 6.71 (m, 2H), 6.69 – 6.64 (m, 2H), 4.39 (br. s, 2H), 4.07 (s, 3H). ^13^C NMR (75 MHz, CDCl_3_) *δ* (ppm) 161.79, 153.55, 138.86, 135.95, 135.16, 129.71, 129.53, 129.44, 128.95, 128.51, 123.68, 122.57, 105.70, 53.09. Exact mass (HRMS, ESI) calculated for C_18_H_15_N_7_O_2_ (M+H)^+^: 362.1360, found 362.1360.

**1,8-Diphenyl-4,8-dihydrobis([1,2,3]triazolo)[4,5-b:4',5'-d]pyridin-5(1*H*)-one (15)**

To a screw-capped reaction tube equipped with a magnetic stirring bar, **14** (32 mg, 0.88 mmol, 1 equiv) and 1 mL HCl (37 %) was heated to 80 °C for 30 minutes. The reaction was cooled to room temperature, diluted with water, and extracted three times with DCM. The organic layer was dried over MgSO_4_, filtered, and concentrated under reduced pressure to obtain compound **15** in 76 % yield (22 mg).

White solid – Mp > 300 °C. ^1^H NMR (400 MHz, DMSO-*d_6_*) *δ* (ppm) 12.96 (s, 1H), 7.37 – 7.30 (m, 4H), 7.30 – 7.22 (m, 2H), 7.13 – 7.05 (m, 4H). ^13^C NMR (101 MHz, DMSO-*d_6_*) *δ* (ppm) 155.30, 147.51, 137.07, 136.51, 135.90, 130.04, 129.83, 128.83, 128.80, 127.86, 124.85, 124.46, 109.32. Exact mass (HRMS, ESI) calculated for C_17_H_11_N_7_O (M+H)^+^: 330.1098, found 330.1090.

**3** (^1^H NMR, 300MHz, CDCl_3_)


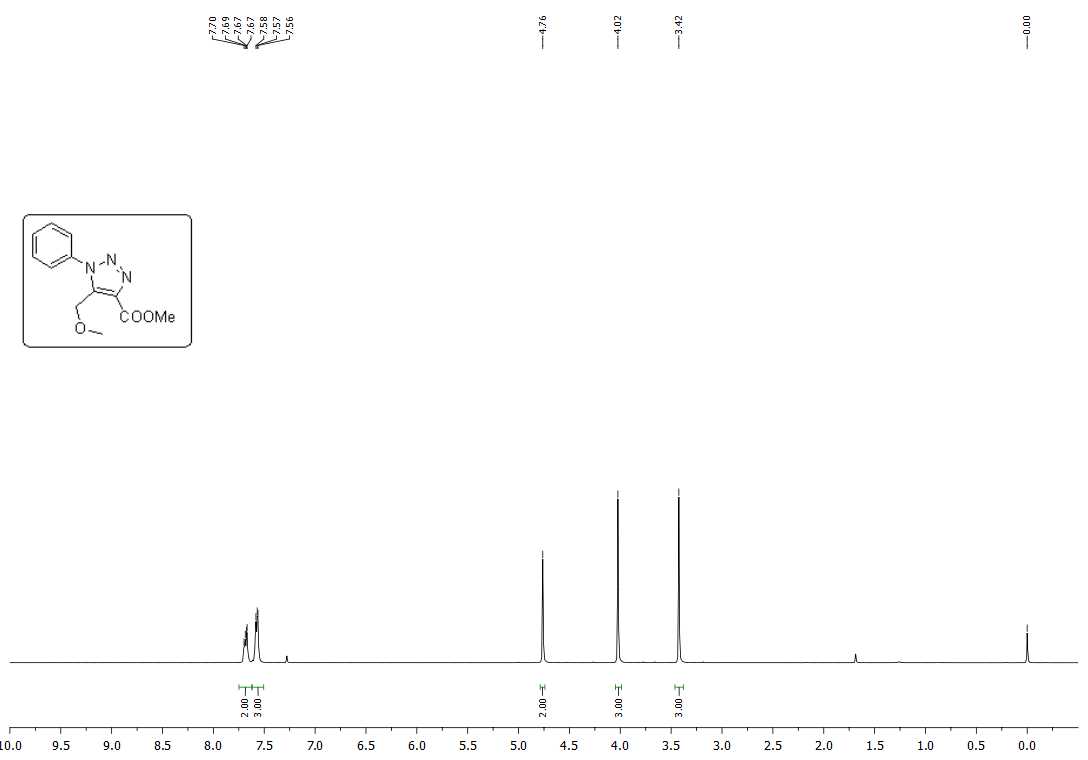


**3** (^13^C NMR, 75 MHz, CDCl_3_)


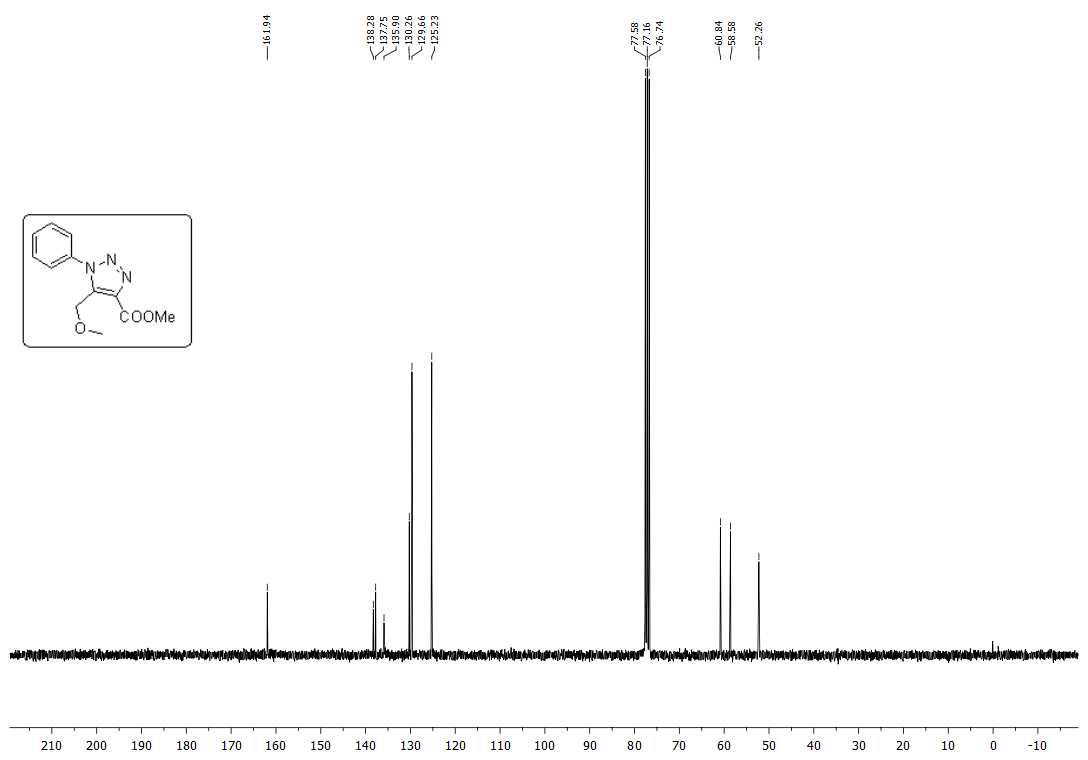


**4** (^1^H NMR, 300MHz, CDCl_3_)

**
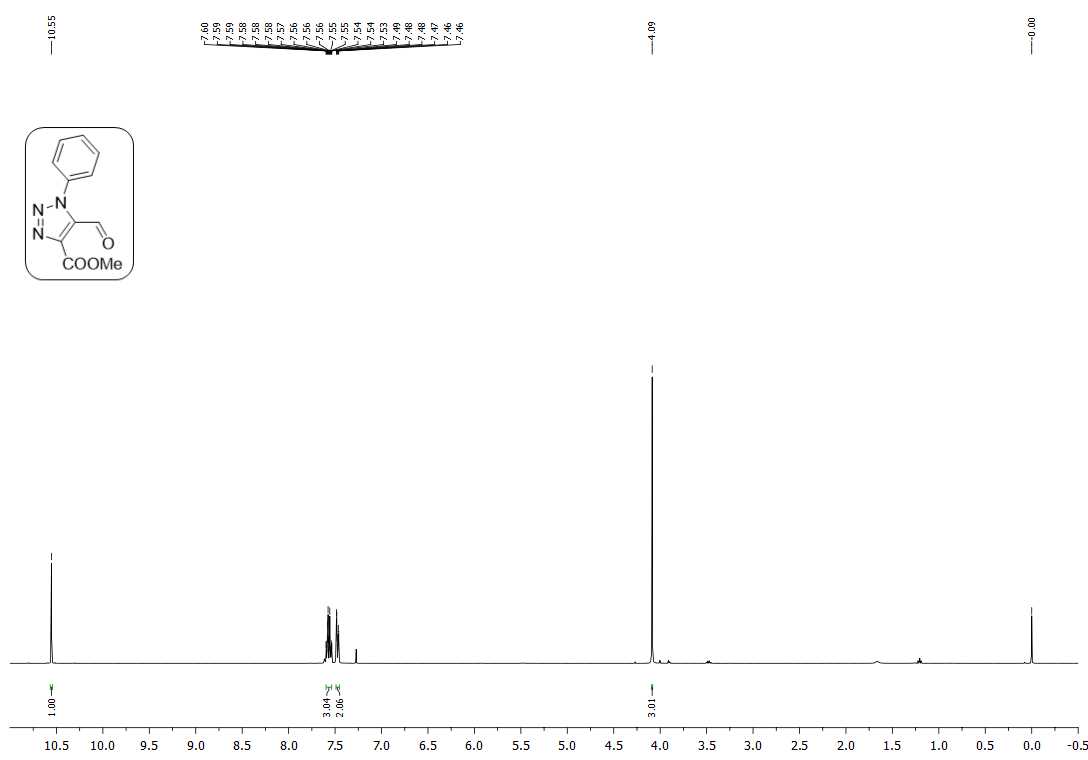
**

**4** (^13^C NMR, 101 MHz, CDCl_3_)


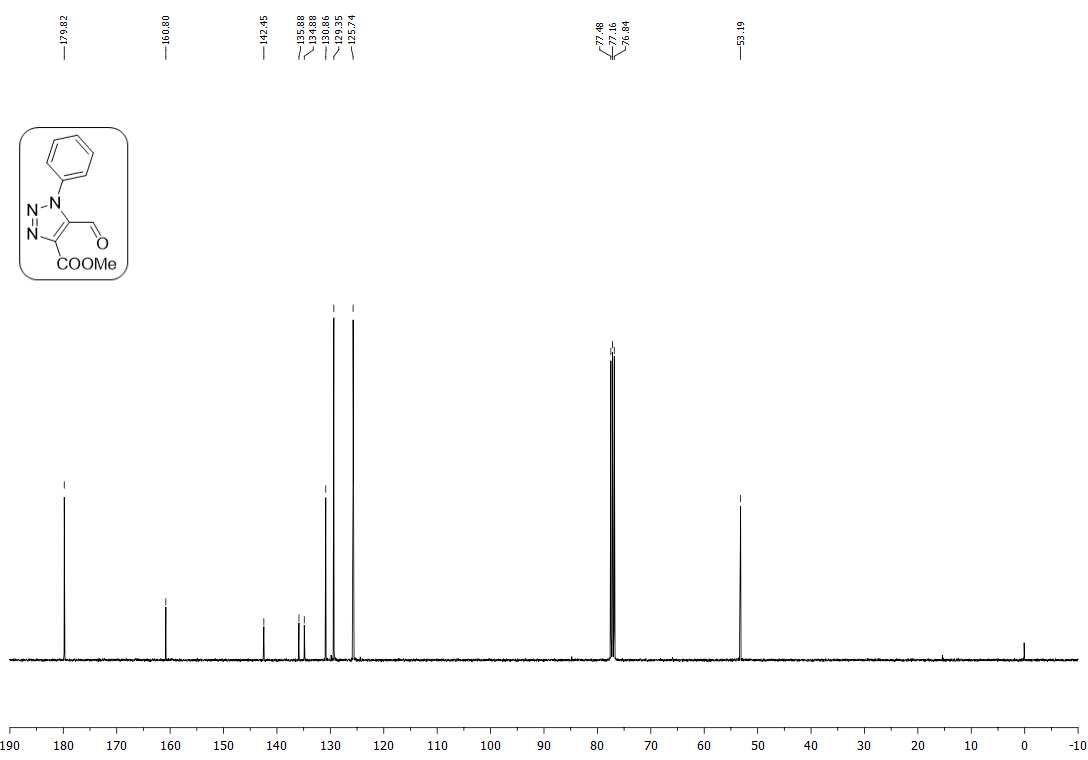


**5** (^1^H NMR, 300 MHz, CDCl_3_)


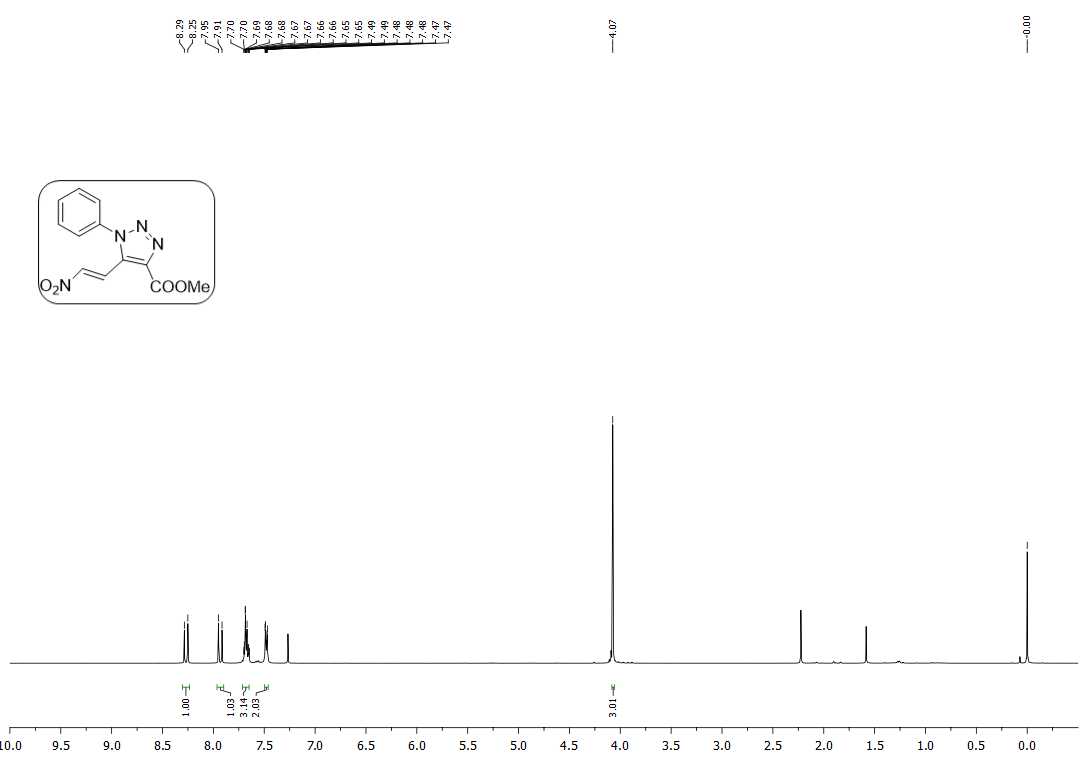


**5** (^13^C NMR, 75 MHz, CDCl_3_)


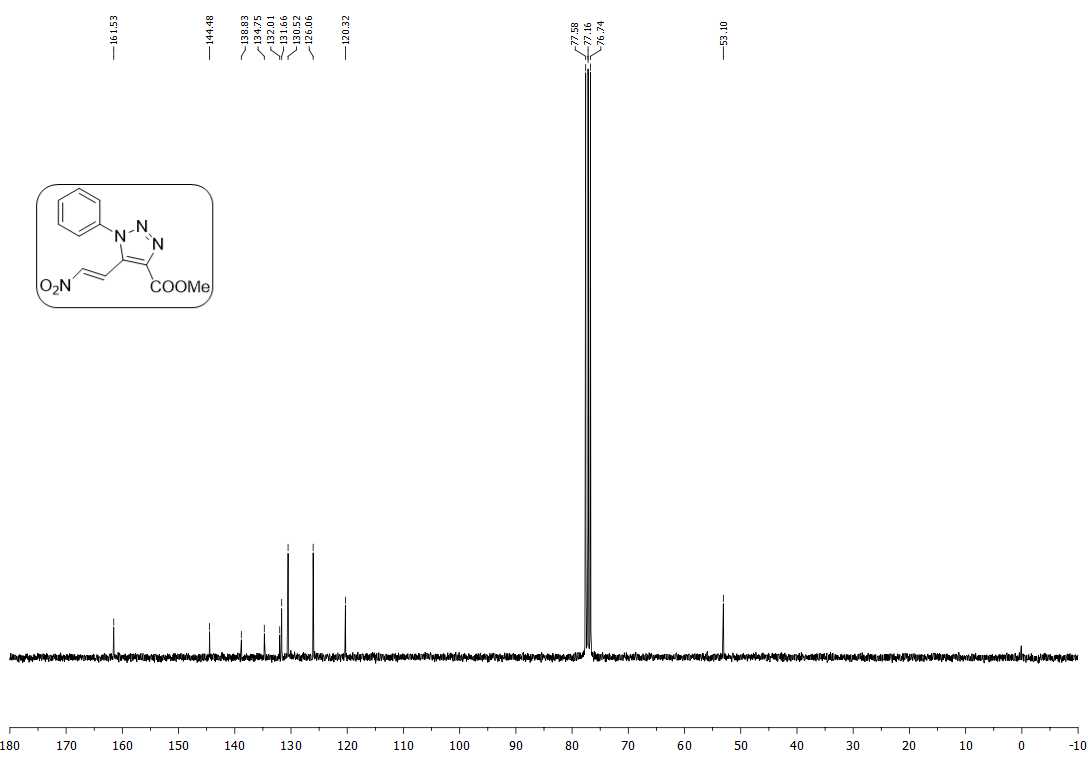


**8a** (^1^H NMR, 400 MHz, CDCl_3_)


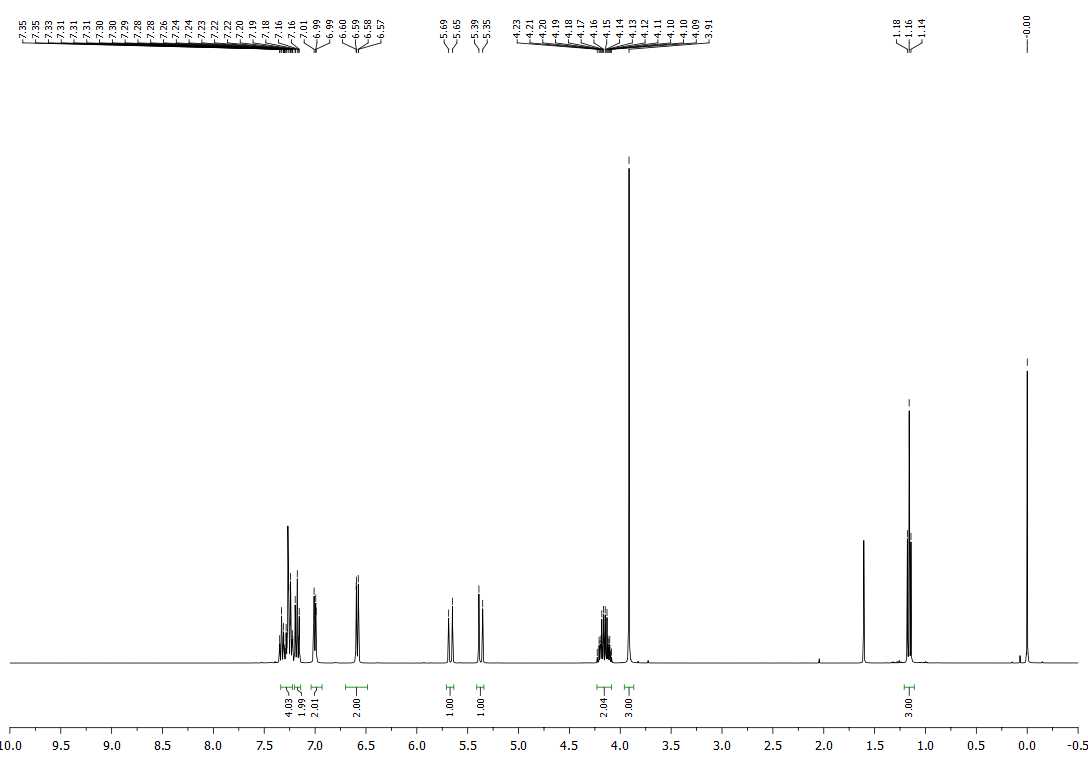


**8a** (^1^H NMR, 600 MHz, Cl_2_CDCDCl_2_)


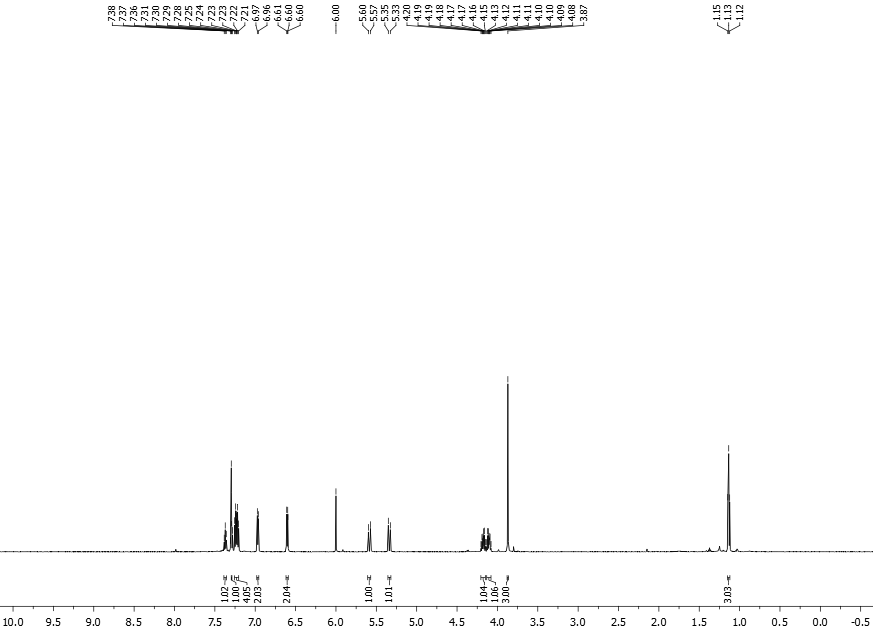


**8a** (^13^C NMR, 101 MHz, CDCl_3_)


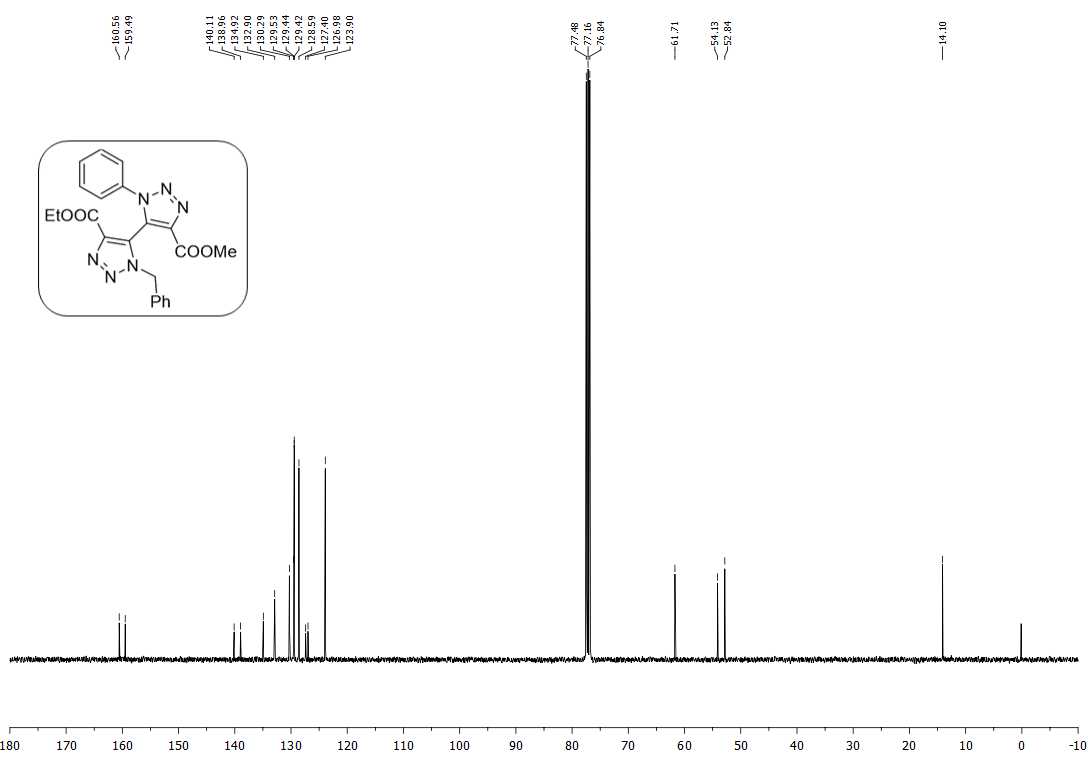


Diastereomer **8ba** (^1^H NMR, 300 MHz, CDCl_3_)


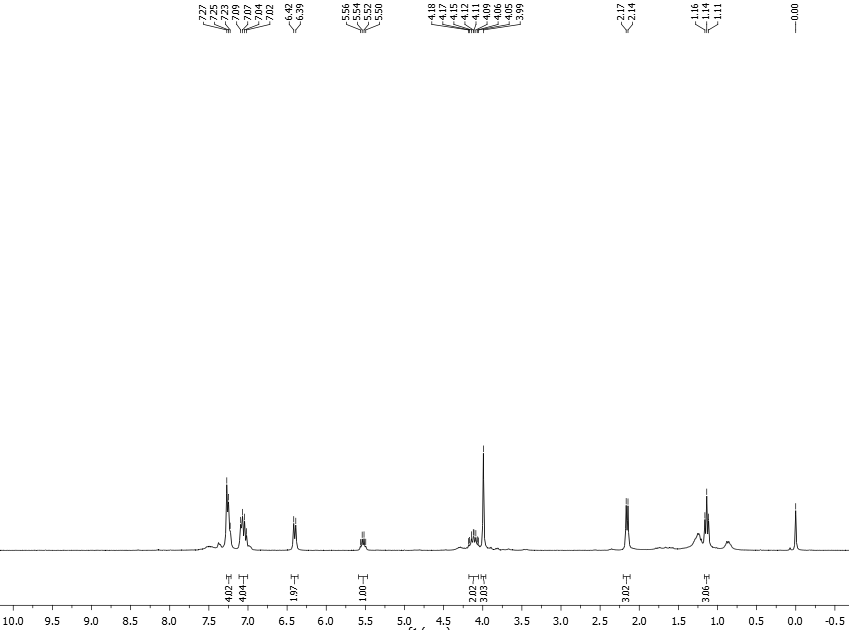


Diastereomer **8ba** (^13^C NMR, 75 MHz, CDCl_3_)


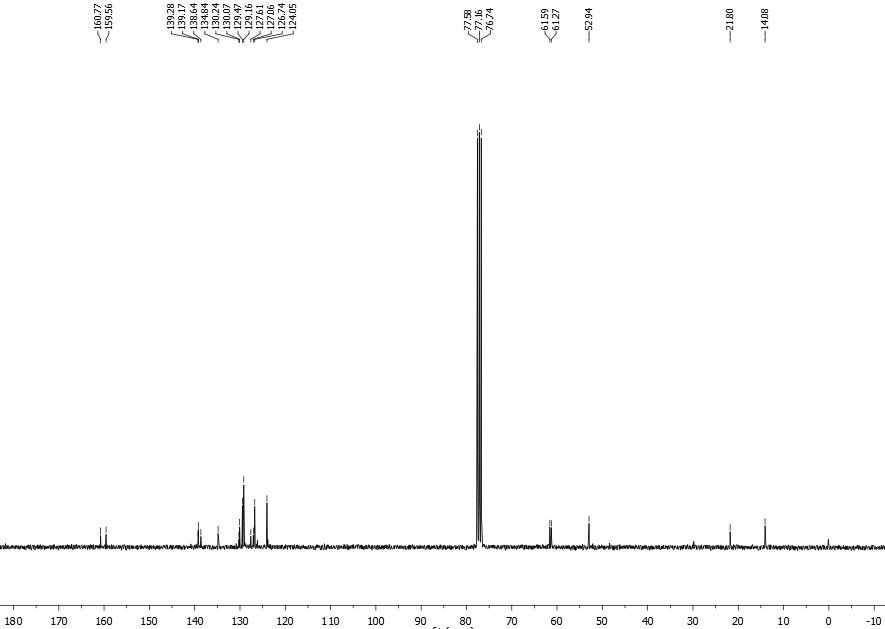


Diastereomer **8bb** (^1^H NMR, 300 MHz, CDCl_3_)


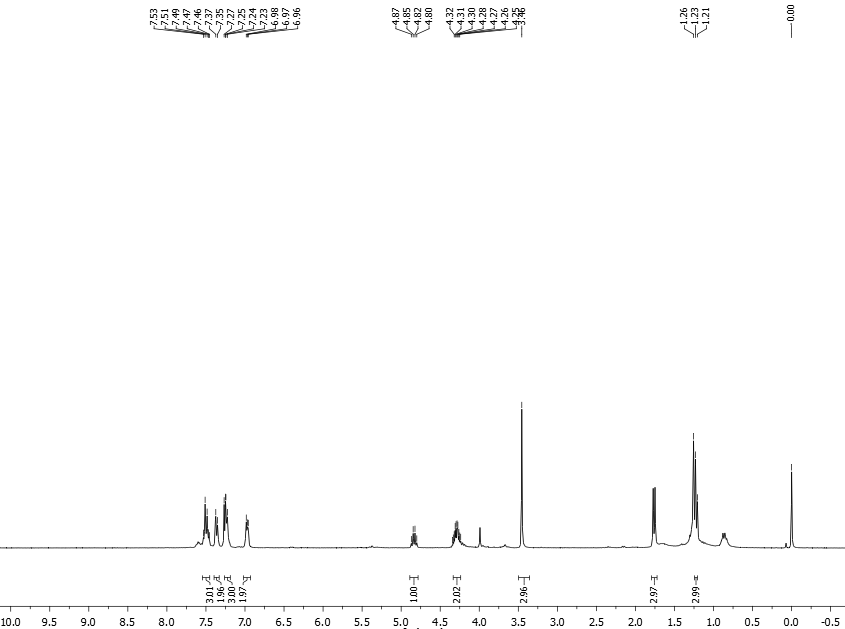


Diastereomer **8bb** (^13^C NMR, 75 MHz, CDCl_3_)


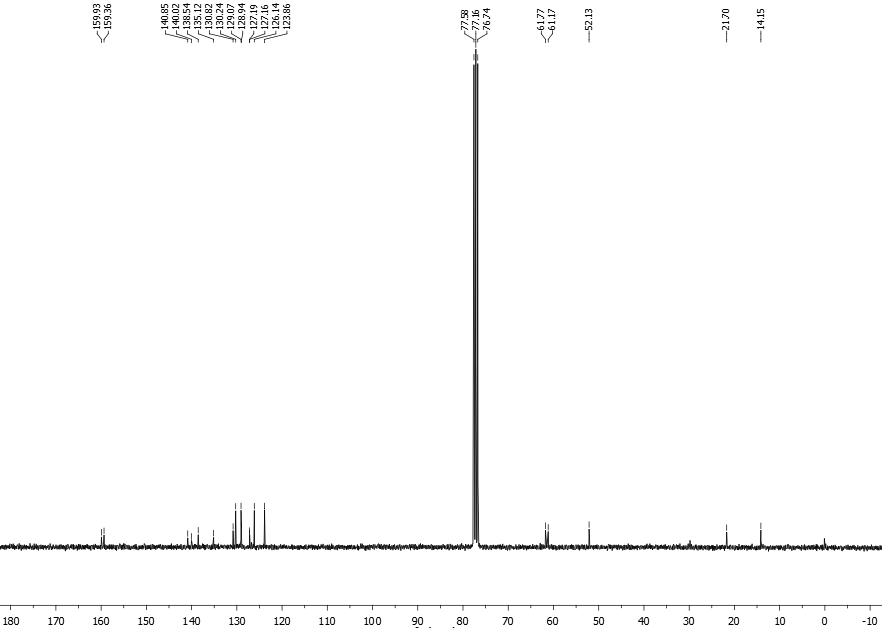


**8c** (^1^H NMR, 300 MHz, CDCl_3_)


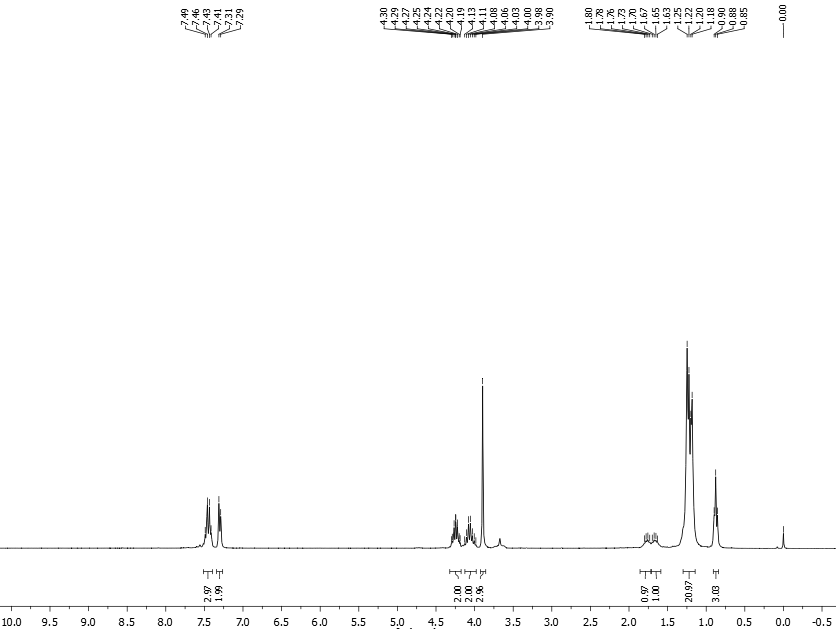


**8c** (^13^C NMR, 75 MHz, CDCl_3_)


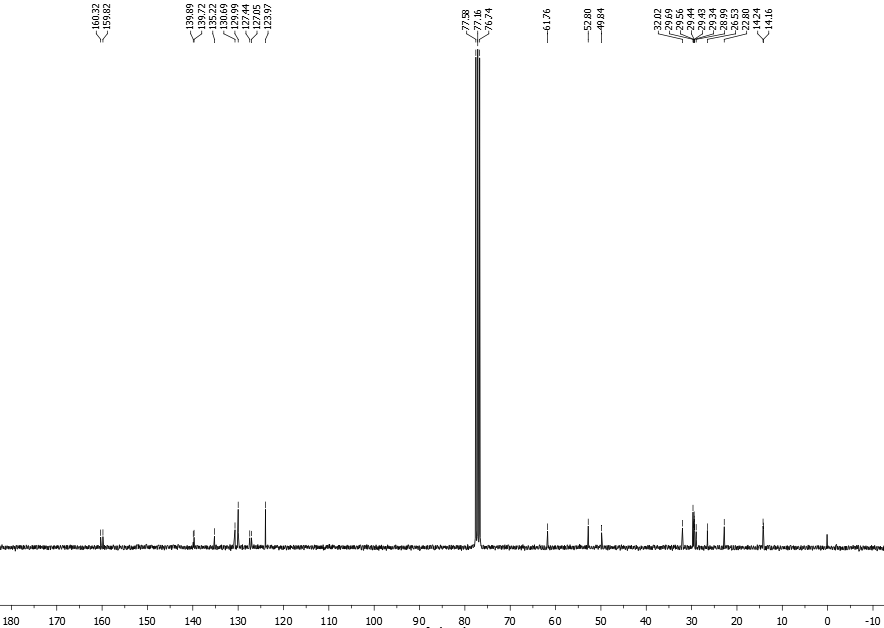


**8d** (^1^H NMR, 300 MHz, CDCl_3_)


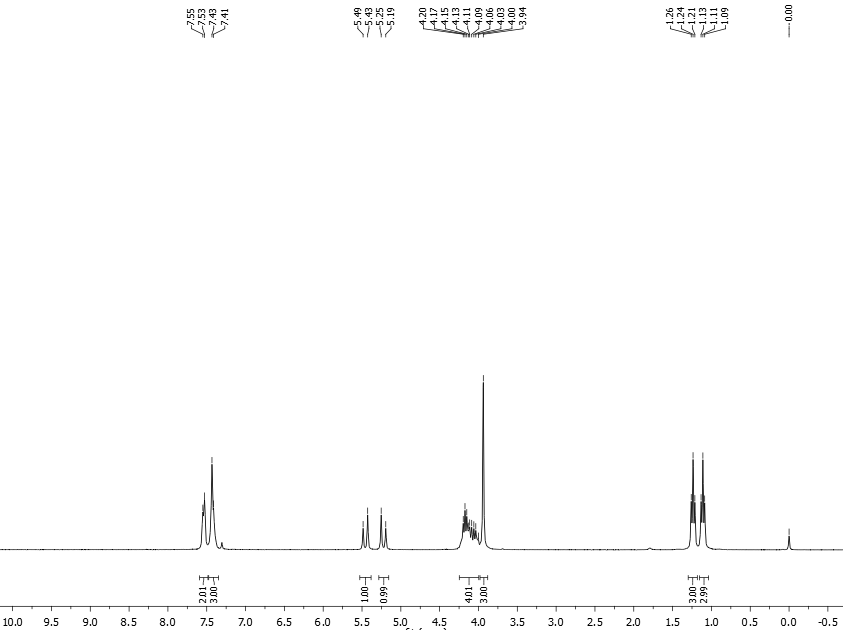


**8d** (^13^C NMR, 75 MHz, CDCl_3_)


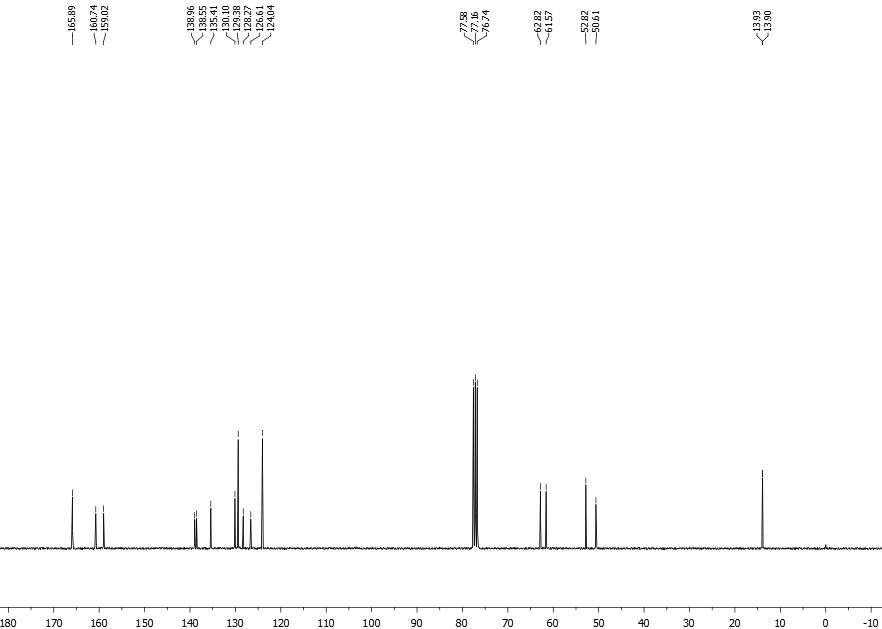


**8e** (^1^H NMR, 300 MHz, CDCl_3_)


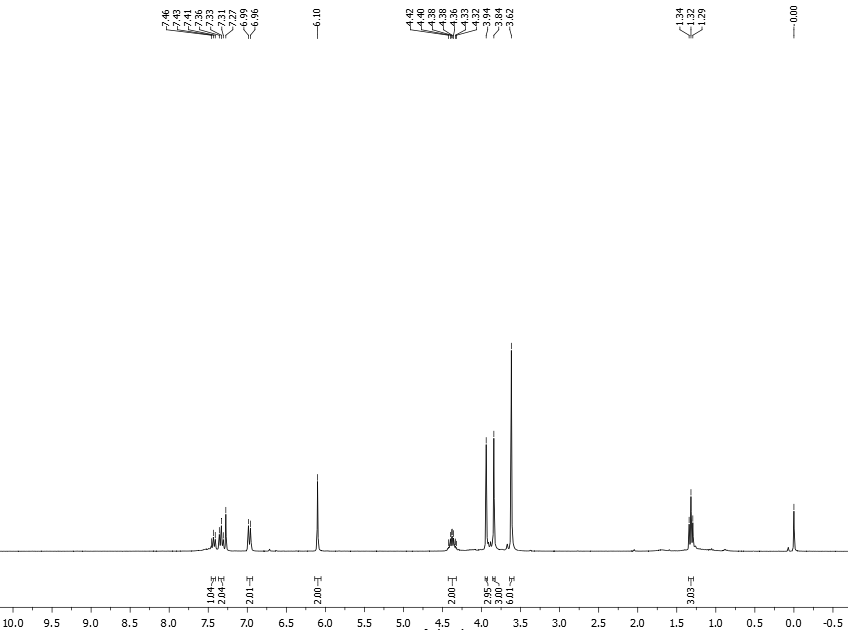


**8e** (^13^C NMR, 75 MHz, CDCl_3_)


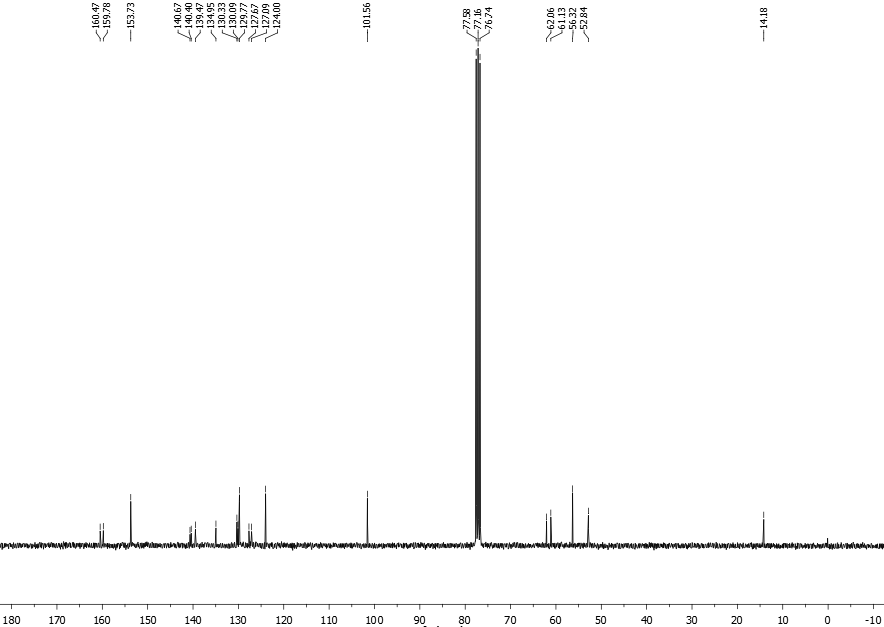


**8f** (^1^H NMR, 300 MHz, CDCl_3_)


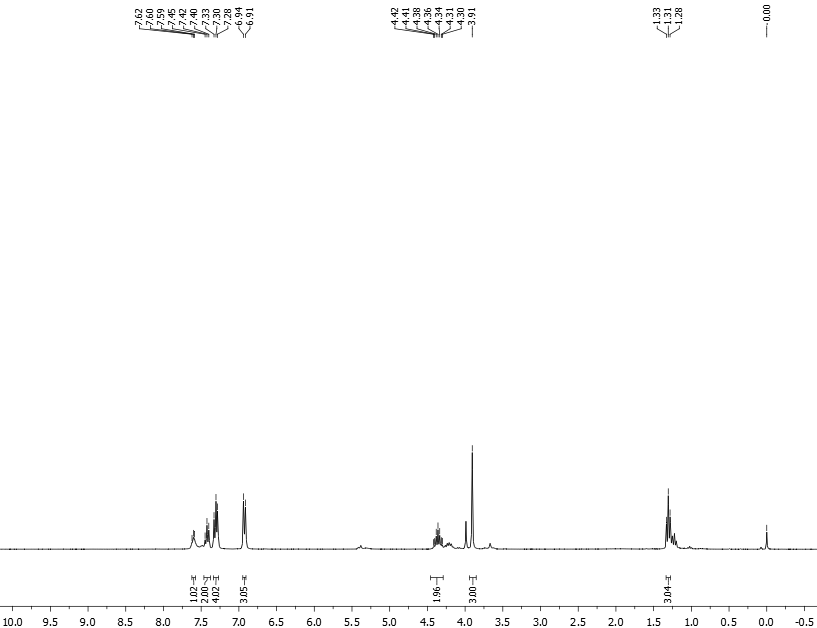


**8f** (^13^C NMR, 75 MHz, CDCl_3_)


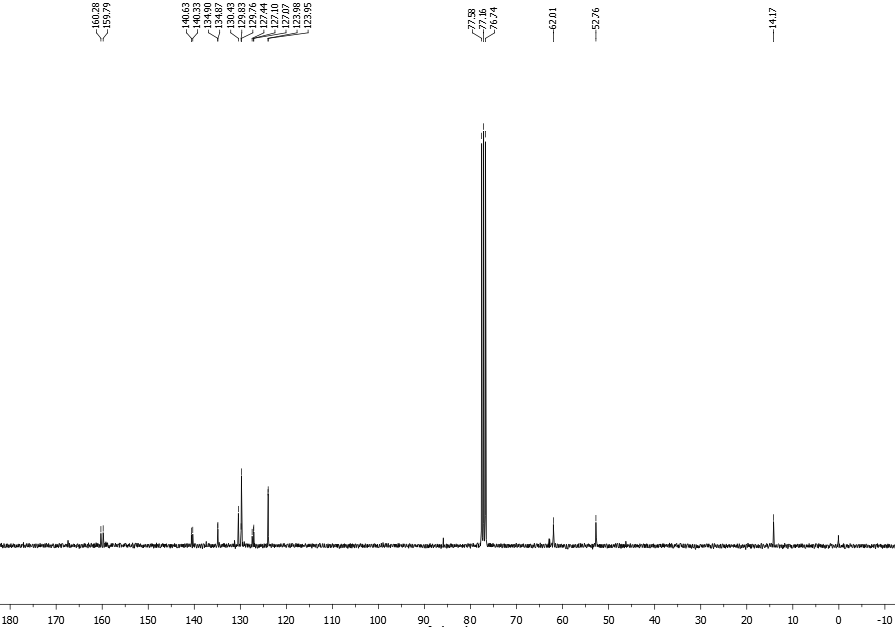


**8g** (^1^H NMR, 300 MHz, CDCl_3_)


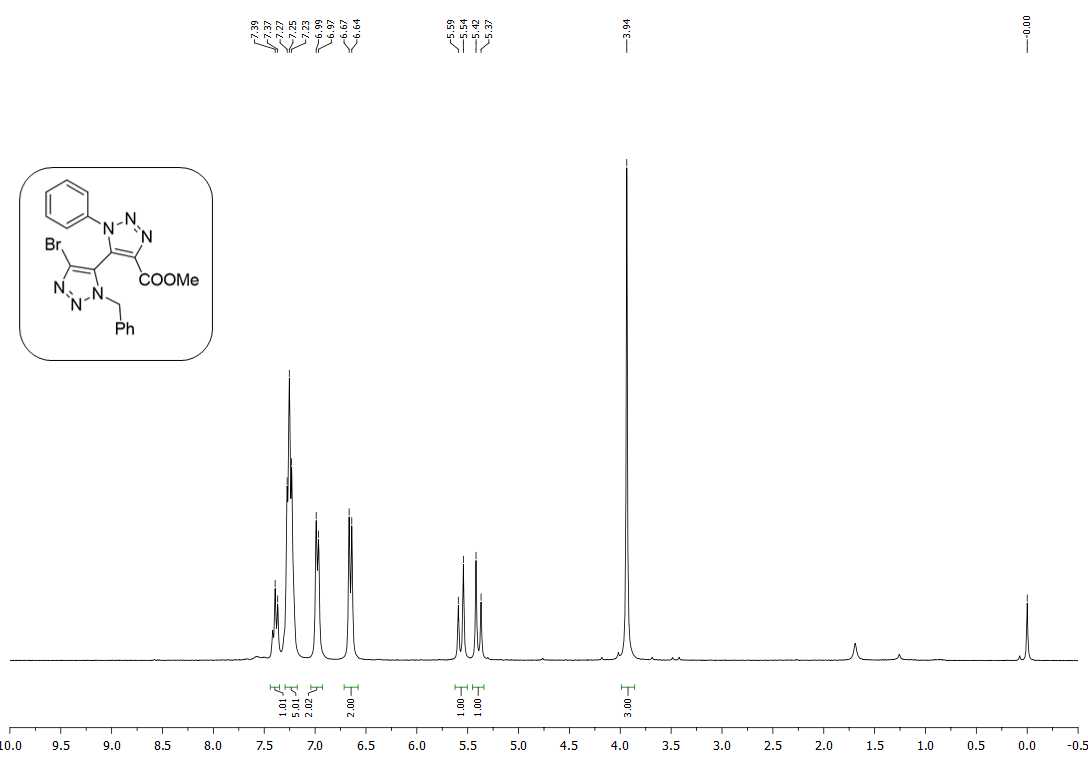


**8g** (^13^C NMR, 75 MHz, CDCl_3_)


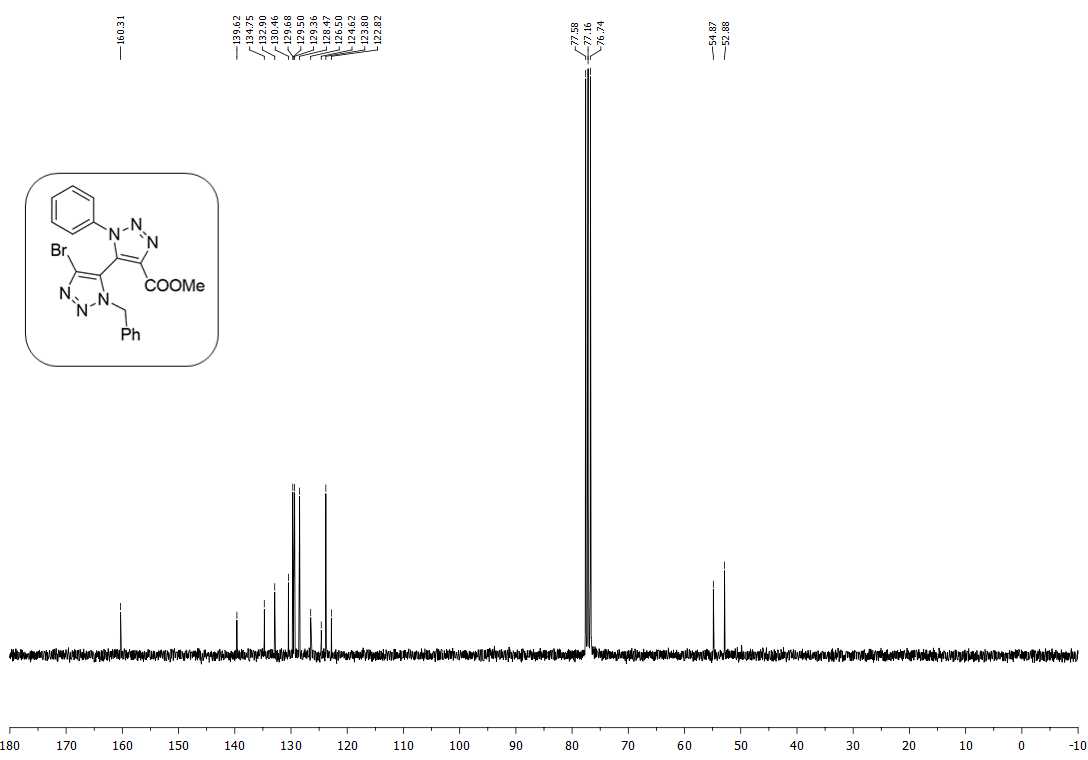


**8h** (^1^H NMR, 300 MHz, CDCl_3_)


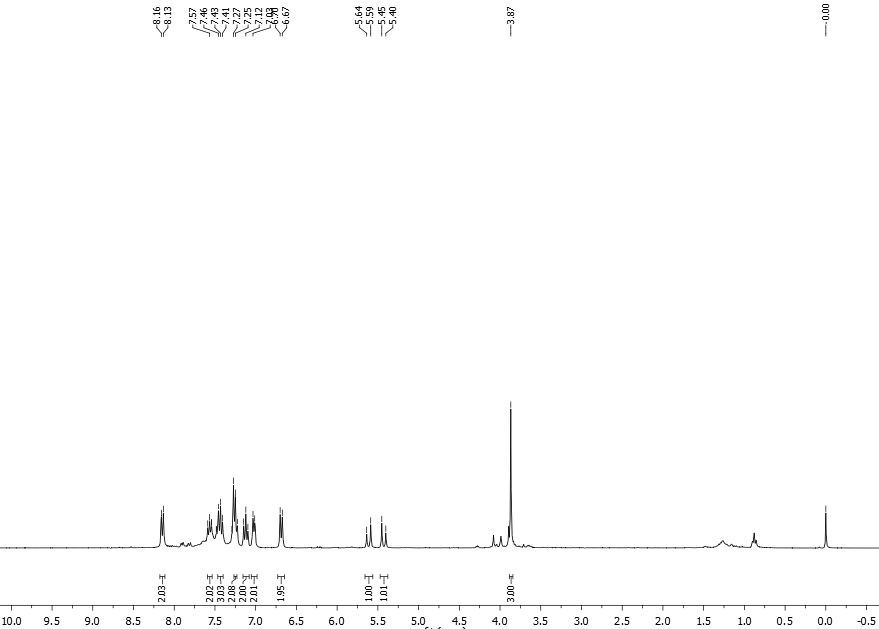


**8h** (^13^C NMR, 75 MHz, CDCl_3_)


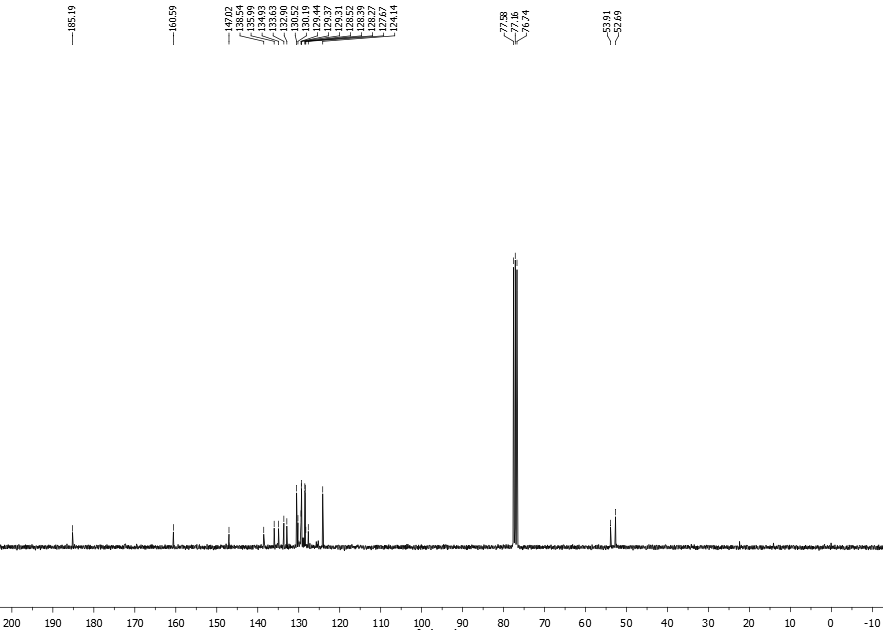


**8i** (^1^H NMR, 300 MHz, CDCl_3_)


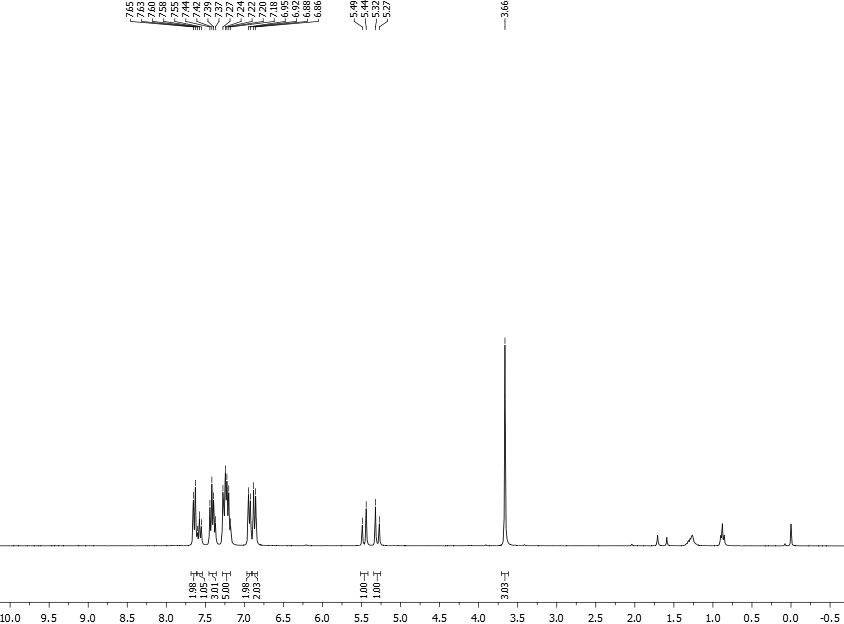


**8i** (^13^C NMR, 75 MHz, CDCl_3_)


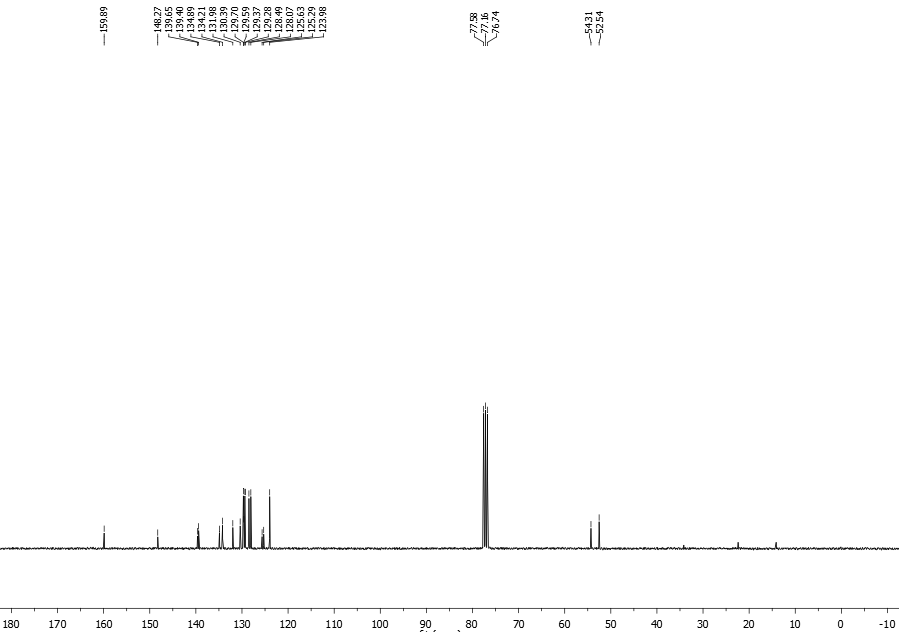


**9a** (^1^H NMR, 400 MHz, CDCl_3_)

**
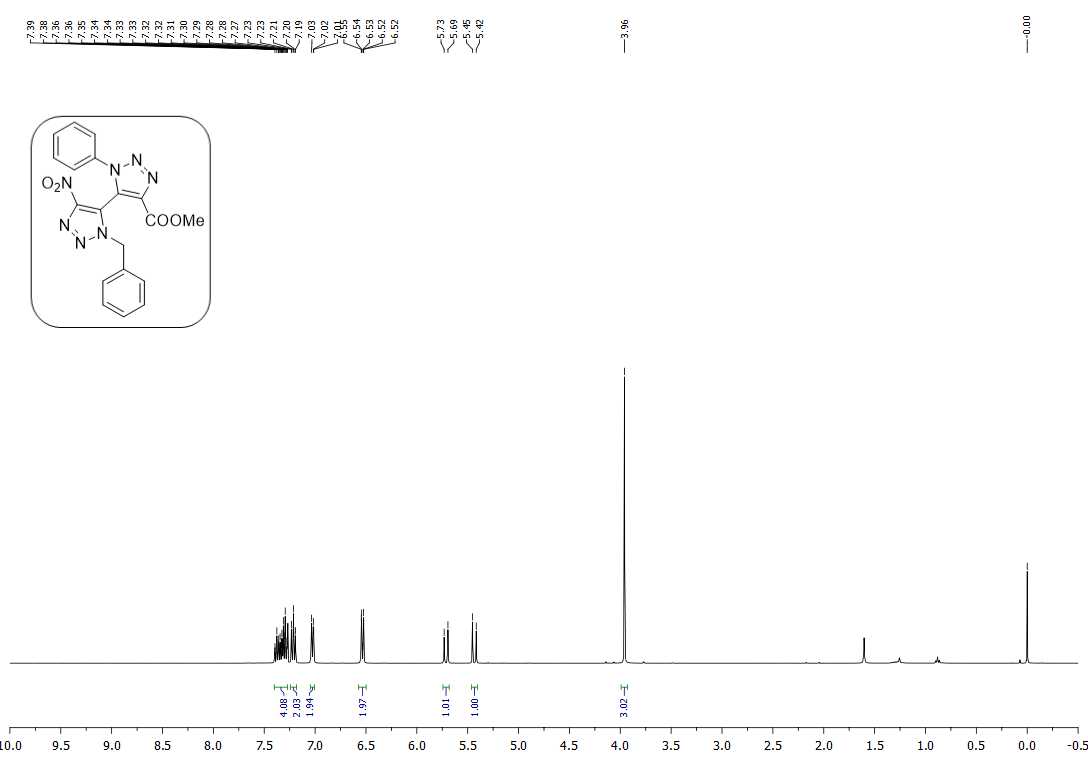
**

**9a** (^1^H NMR, 600 MHz, Cl_2_CDCDCl_2_)


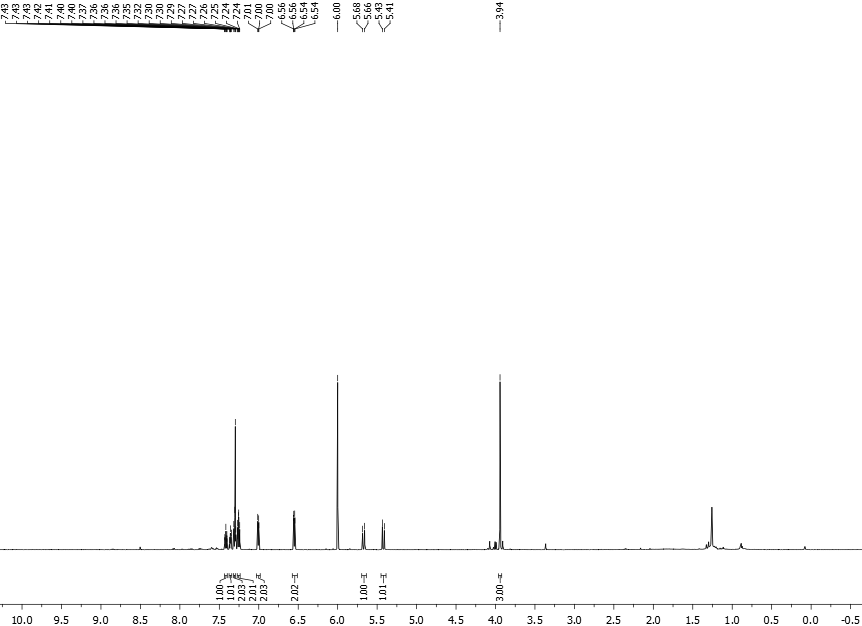


**9a** (^13^C NMR, 101 MHz, CDCl_3_)

**
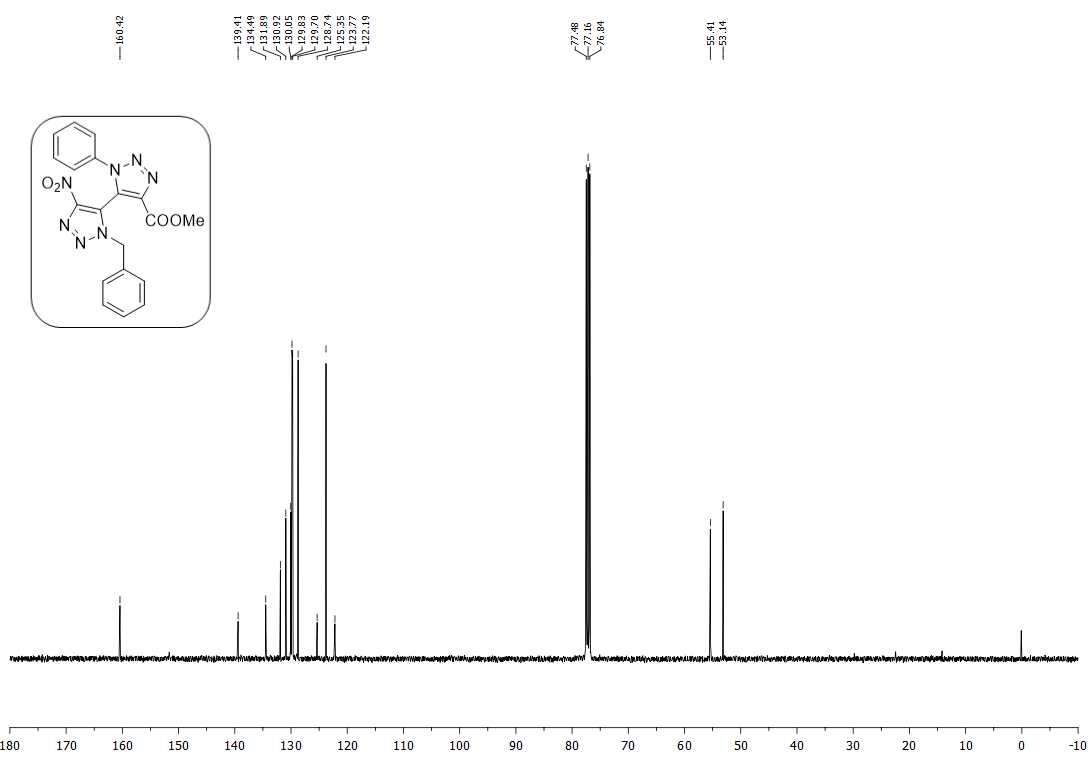
**

**Diastereomer 9ba** (^1^H NMR, 400 MHz, DMSO-*d_6_*)


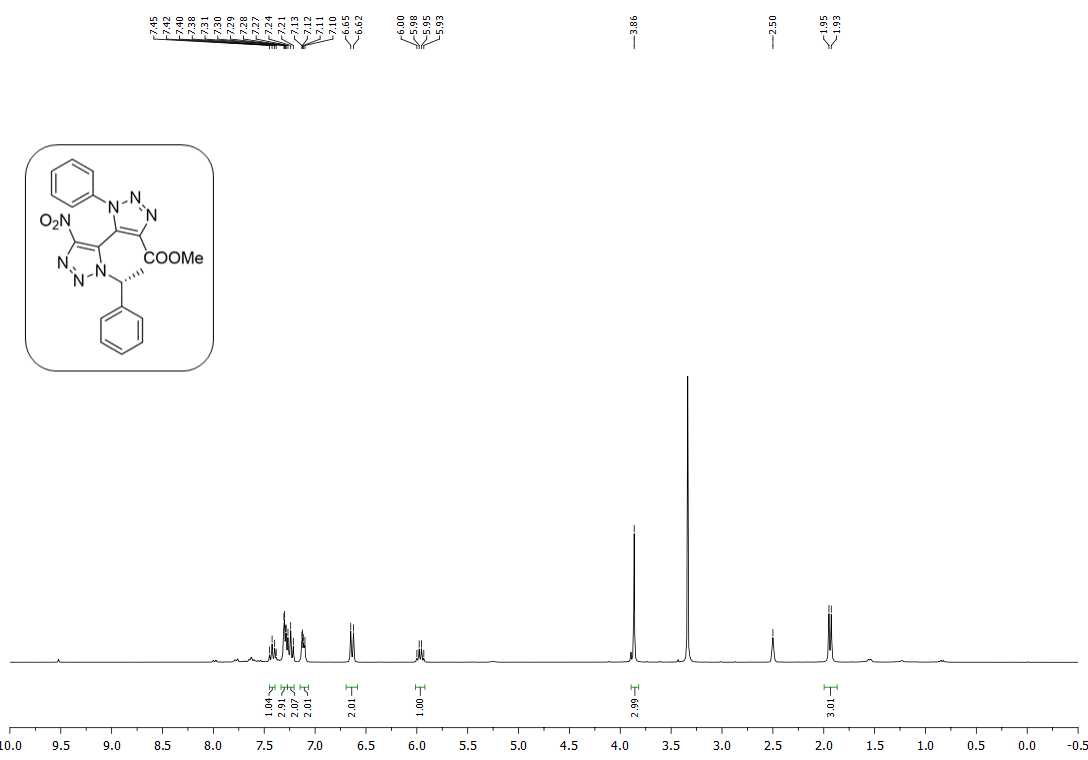


**Diastereomer 9ba** (^13^C NMR, 101 MHz, DMSO-*d_6_*)

**
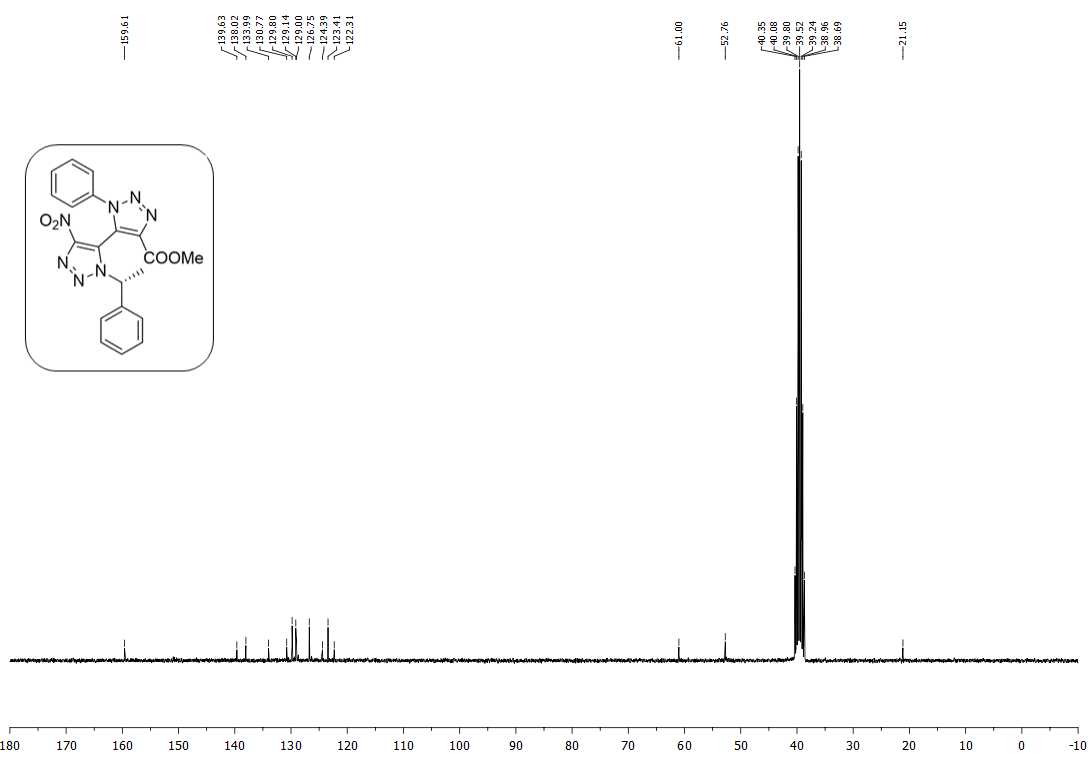
**

**Diastereomer 9bb** (^1^H NMR, 400 MHz, DMSO-*d_6_*)


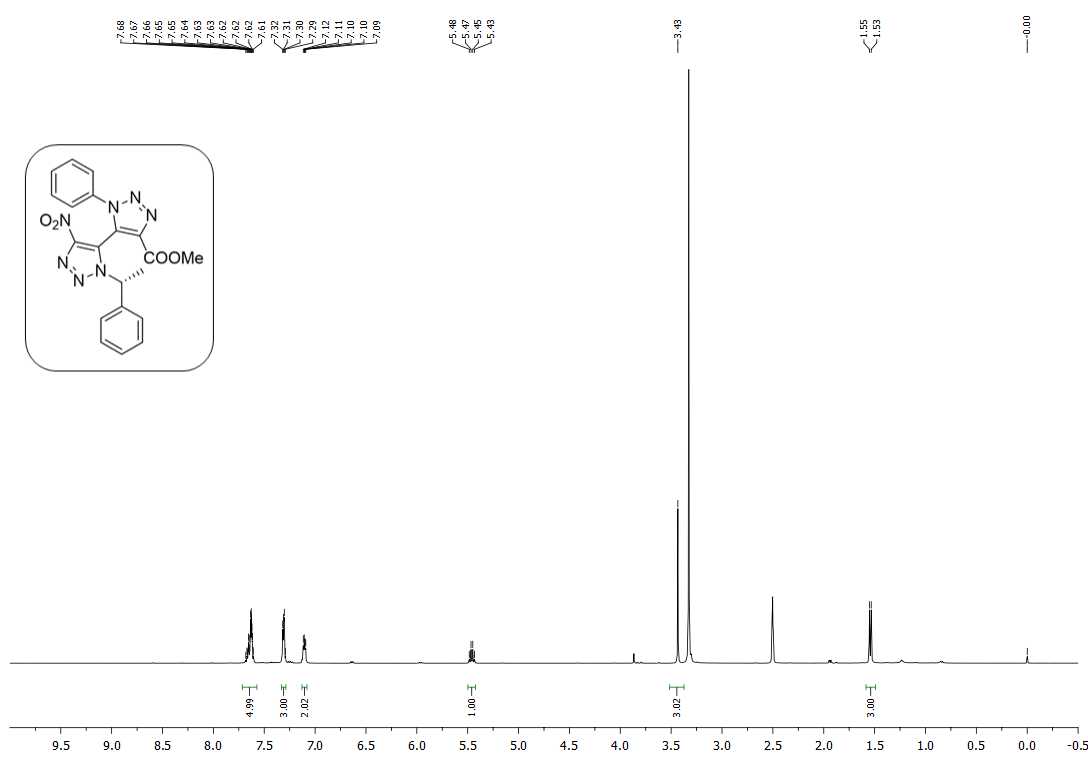


**Diastereomer 9bb** (^13^C NMR, 101 MHz, DMSO-*d_6_*)


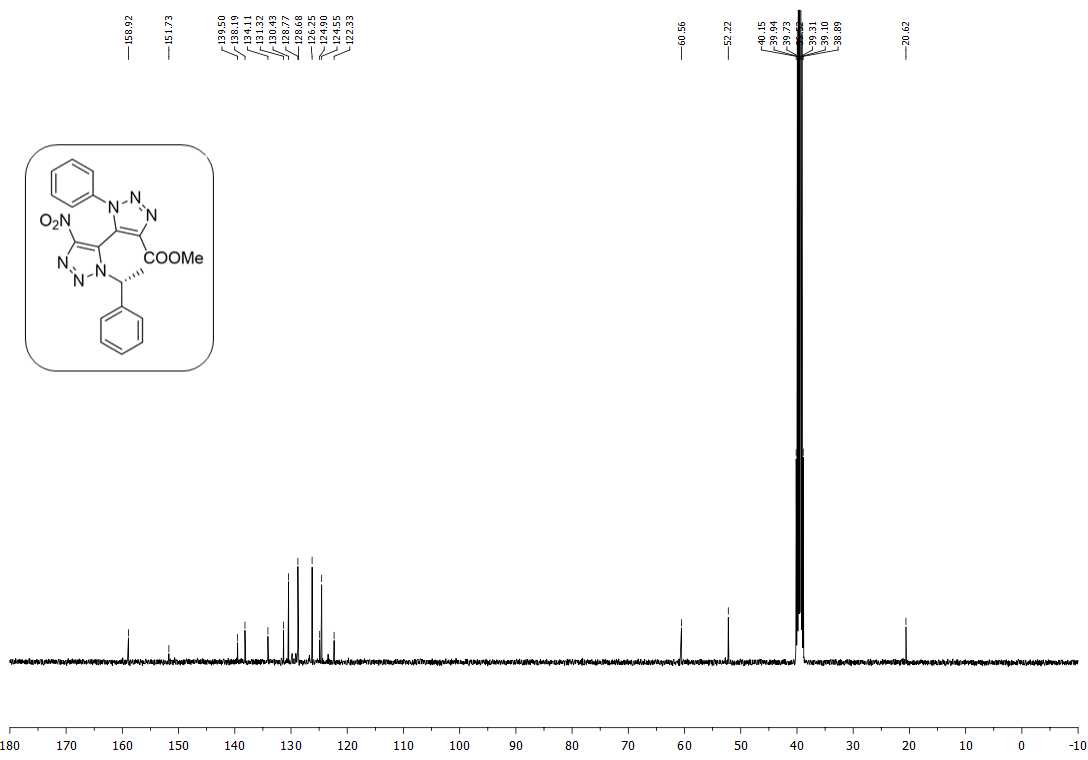


**9c** (^1^H NMR, 300 MHz, CDCl_3_)


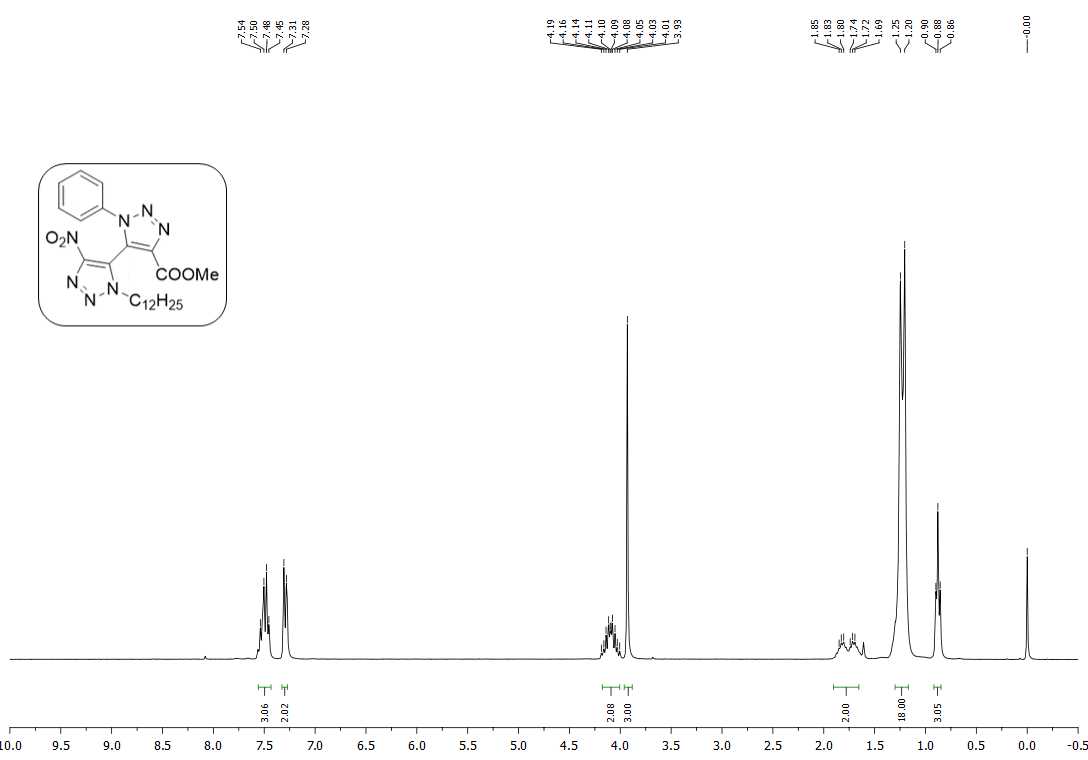


**9c** (^13^C NMR, 75 MHz, CDCl_3_)


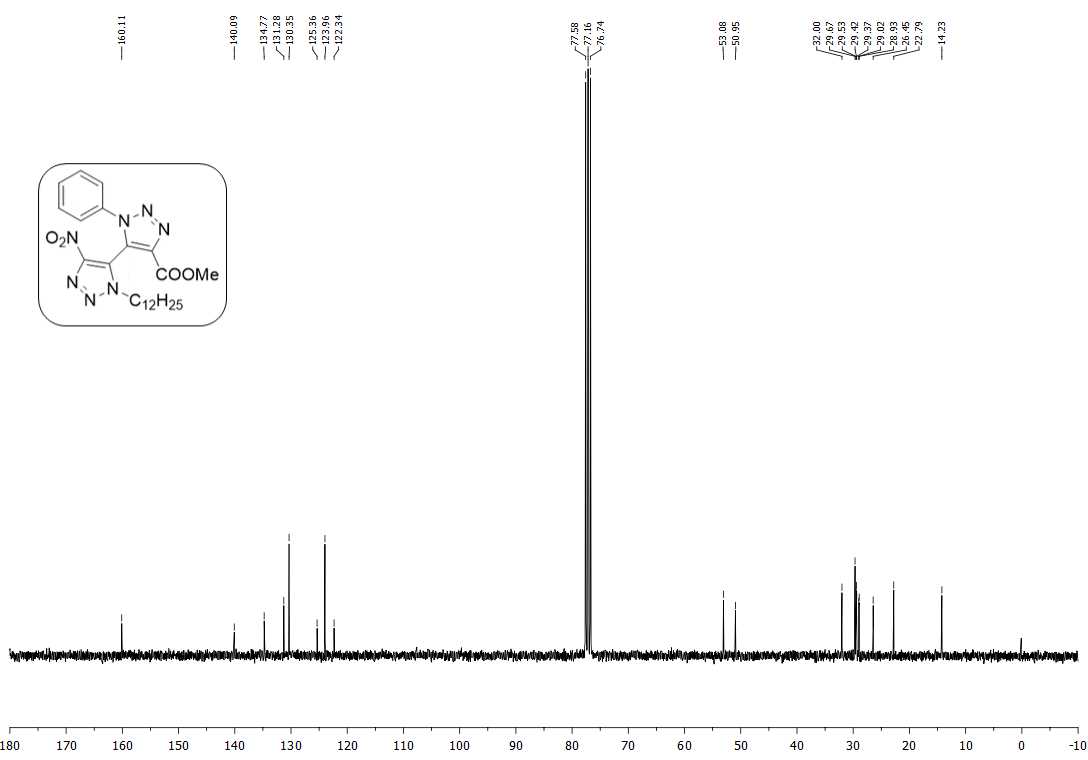


**9d** (^1^H NMR, 400 MHz, CDCl_3_)


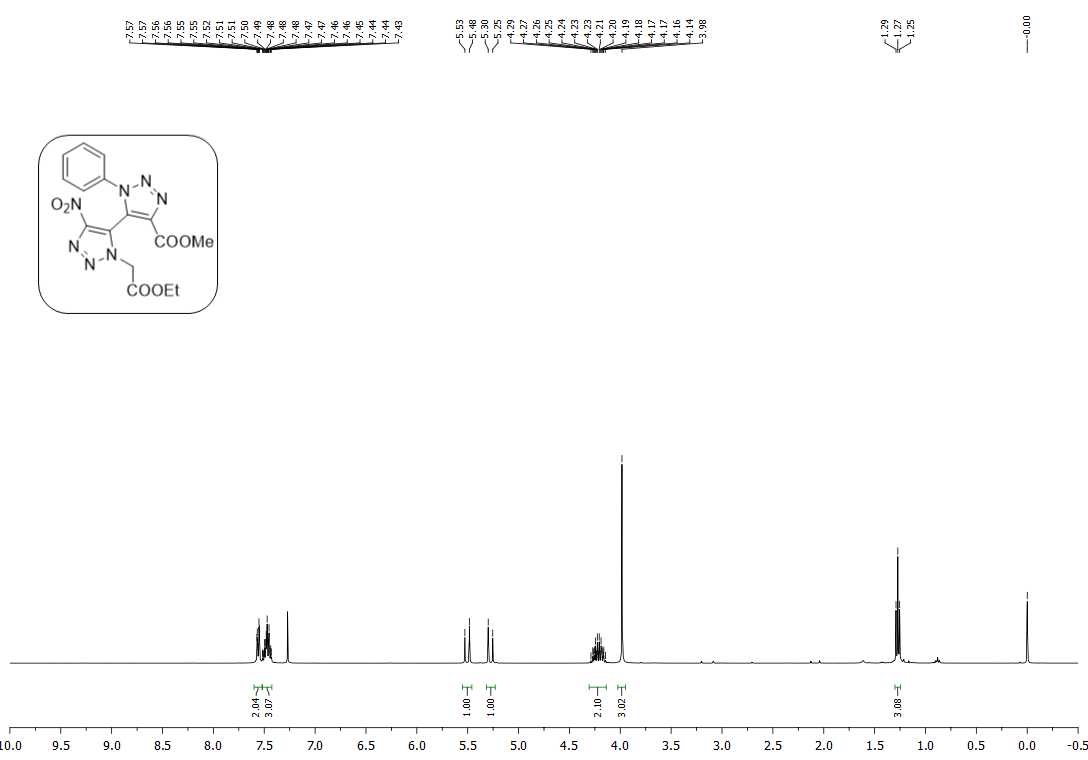


**9d** (^13^C NMR, 101 MHz, CDCl_3_)


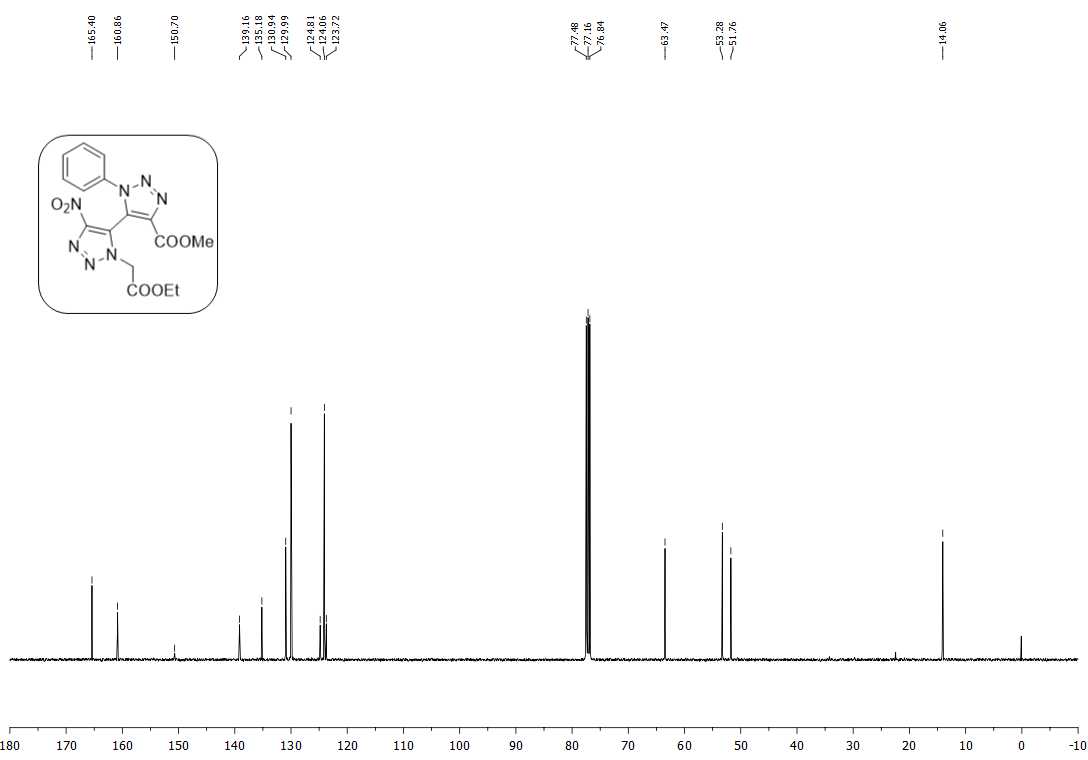


**9e** (^1^H NMR, 400 MHz, CDCl_3_)

**
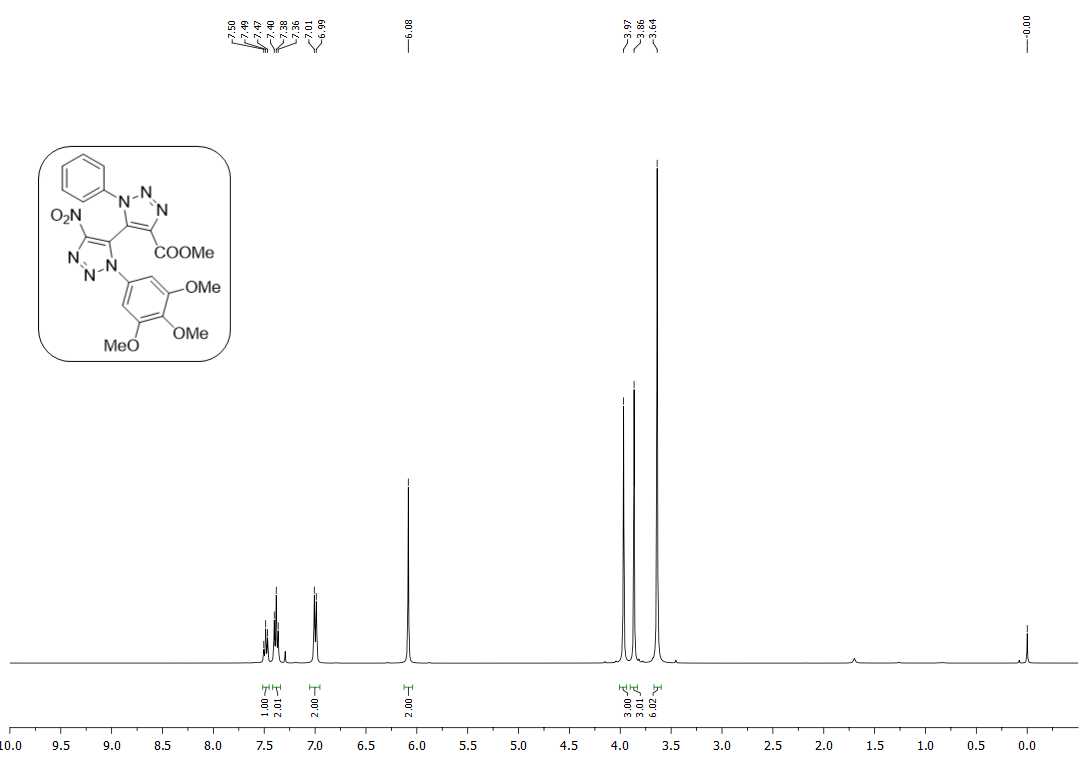
**

**9e** (^13^C NMR, 75 MHz, CDCl_3_)


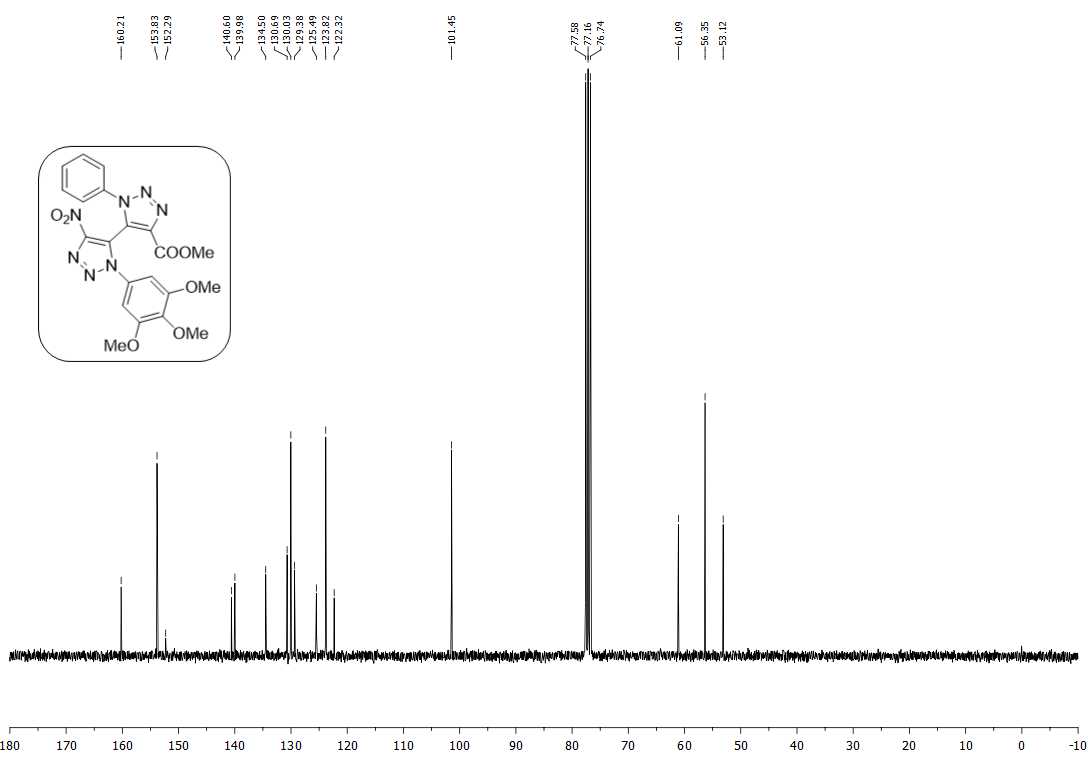


**9f** (^1^H NMR, 300 MHz, CDCl_3_)


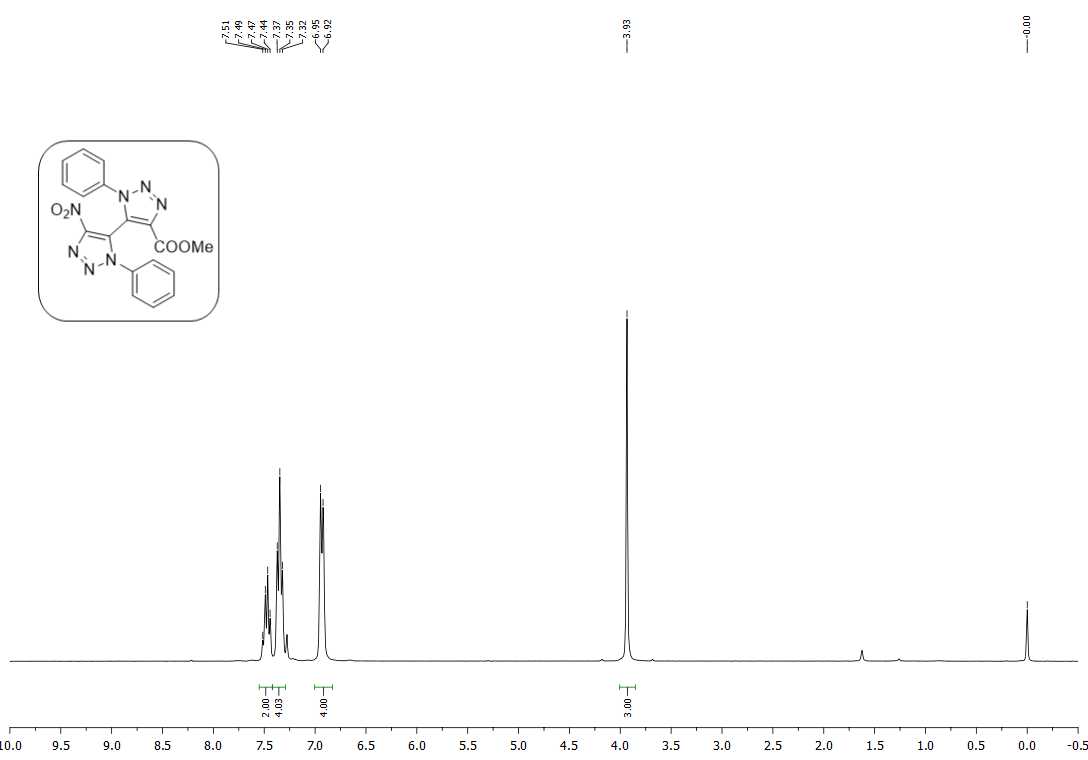


**9f** (^13^C NMR, 75 MHz, CDCl_3_)

**
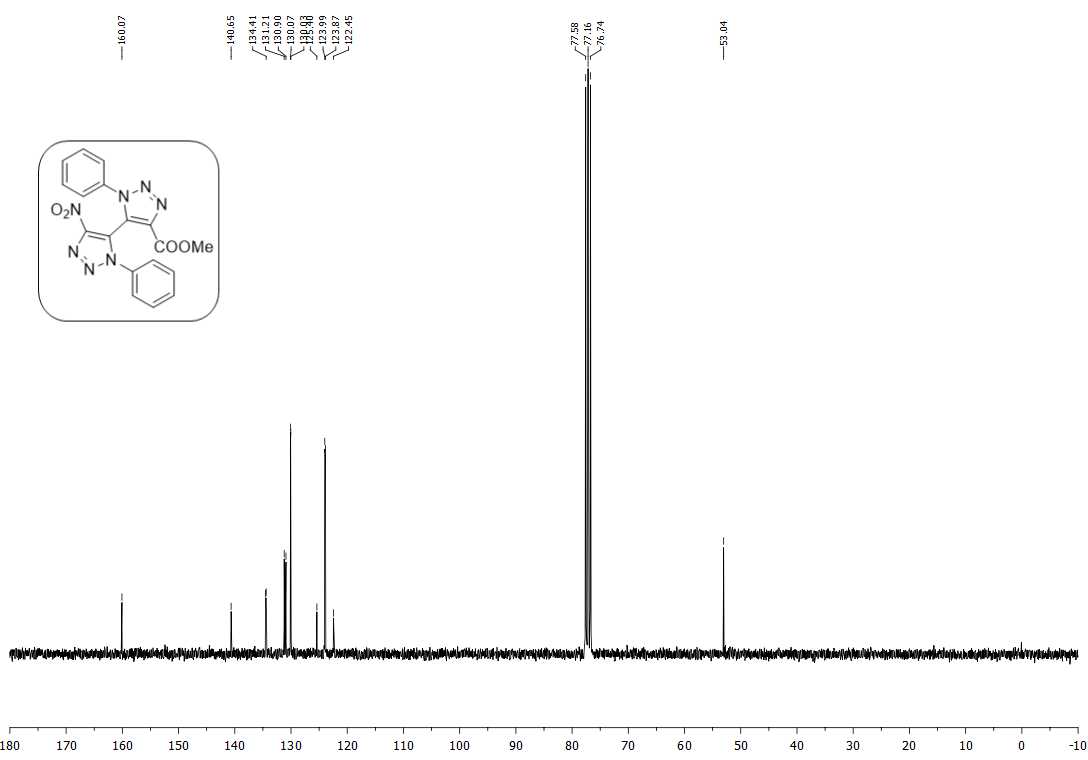
**

**10** (^1^H NMR, 400 MHz, CDCl_3_)


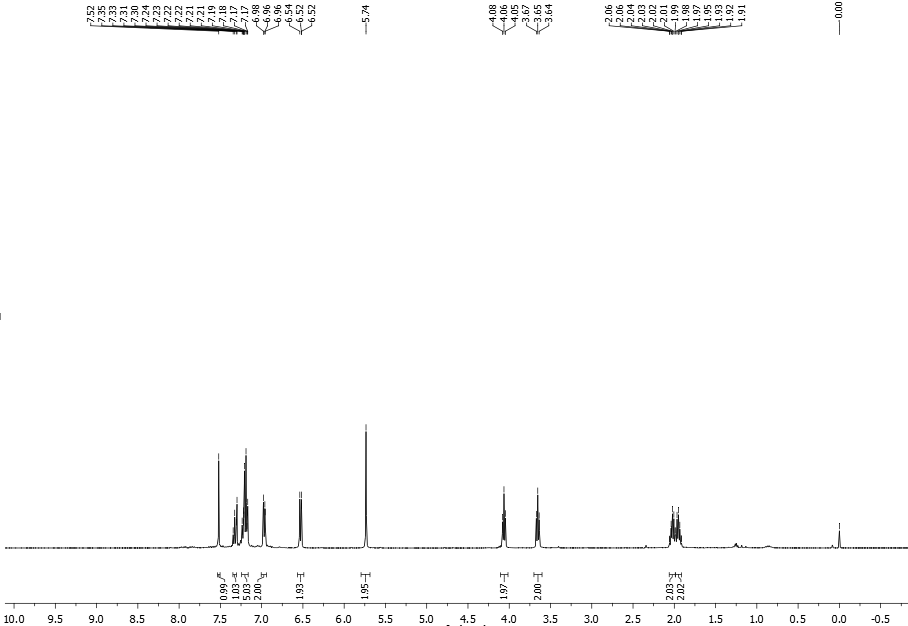


**10** (^13^C NMR, 101 MHz, CDCl_3_)


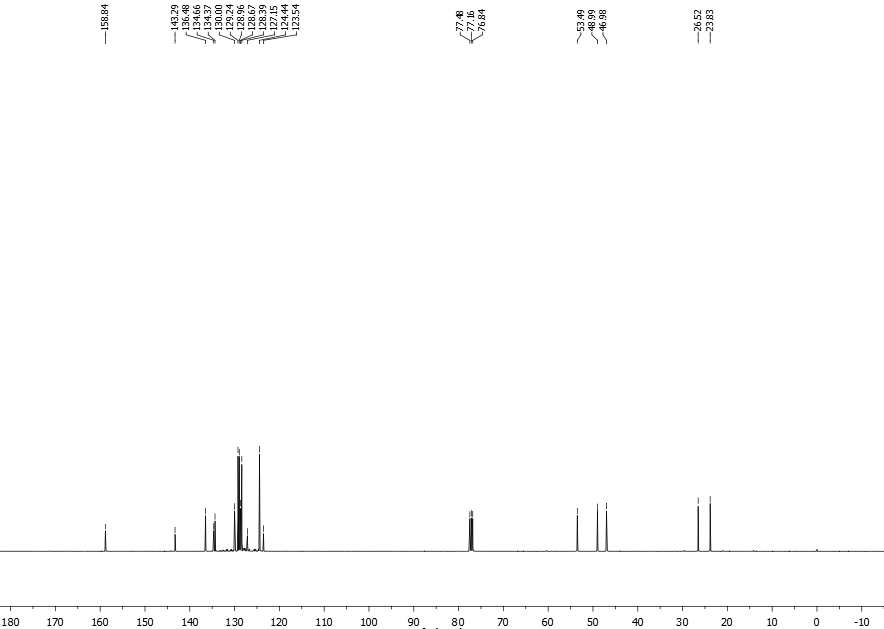


**11** (^1^H NMR, 400 MHz, CDCl_3_)


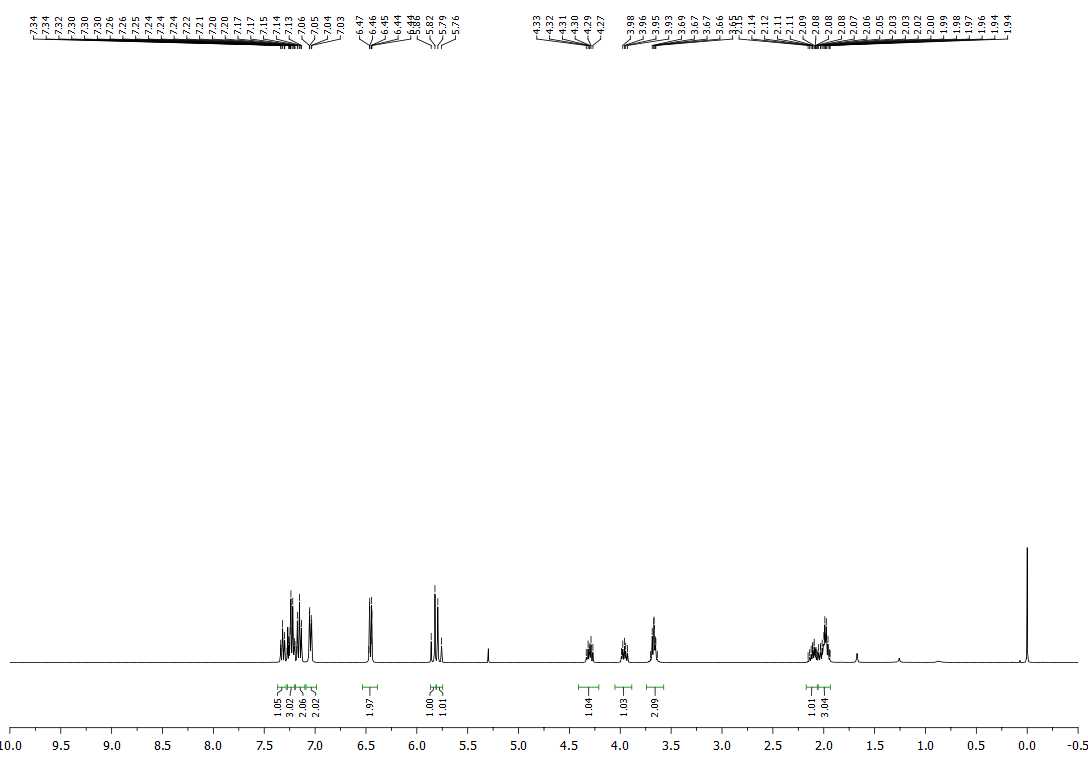


**11** (^13^C NMR, 101 MHz, CDCl_3_)


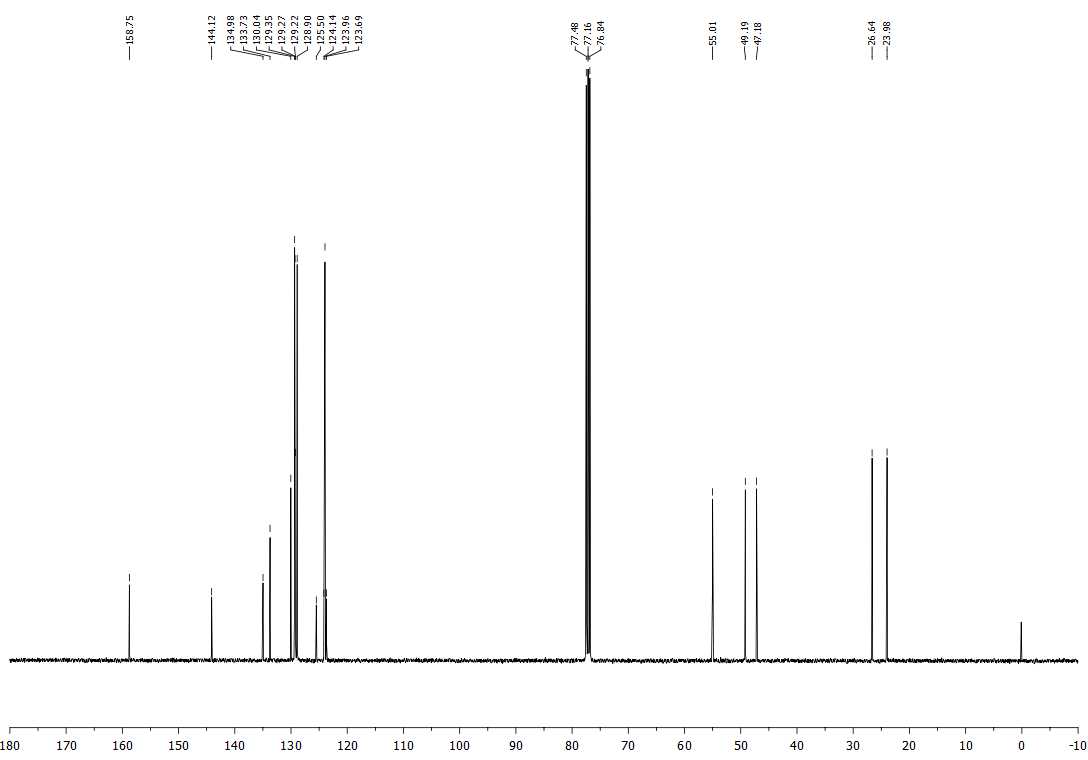


**12** (^1^H NMR, 400 MHz, CDCl_3_)

**
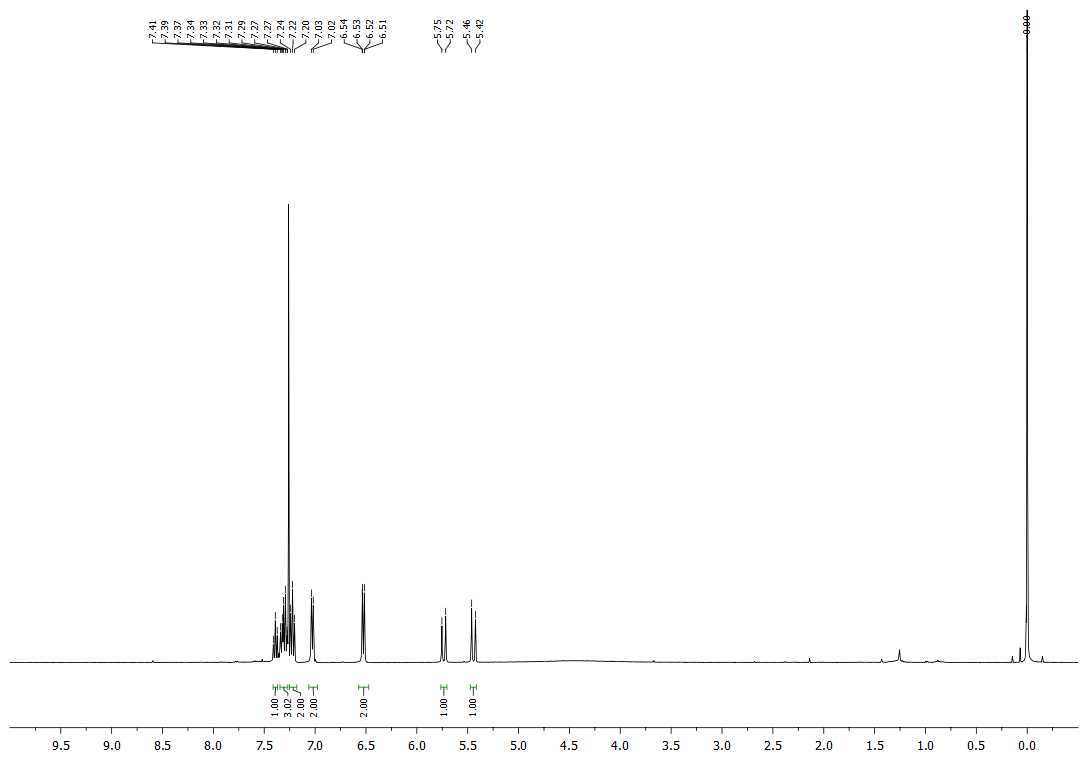
**

**12** (^13^C NMR, 101 MHz, CDCl_3_)**
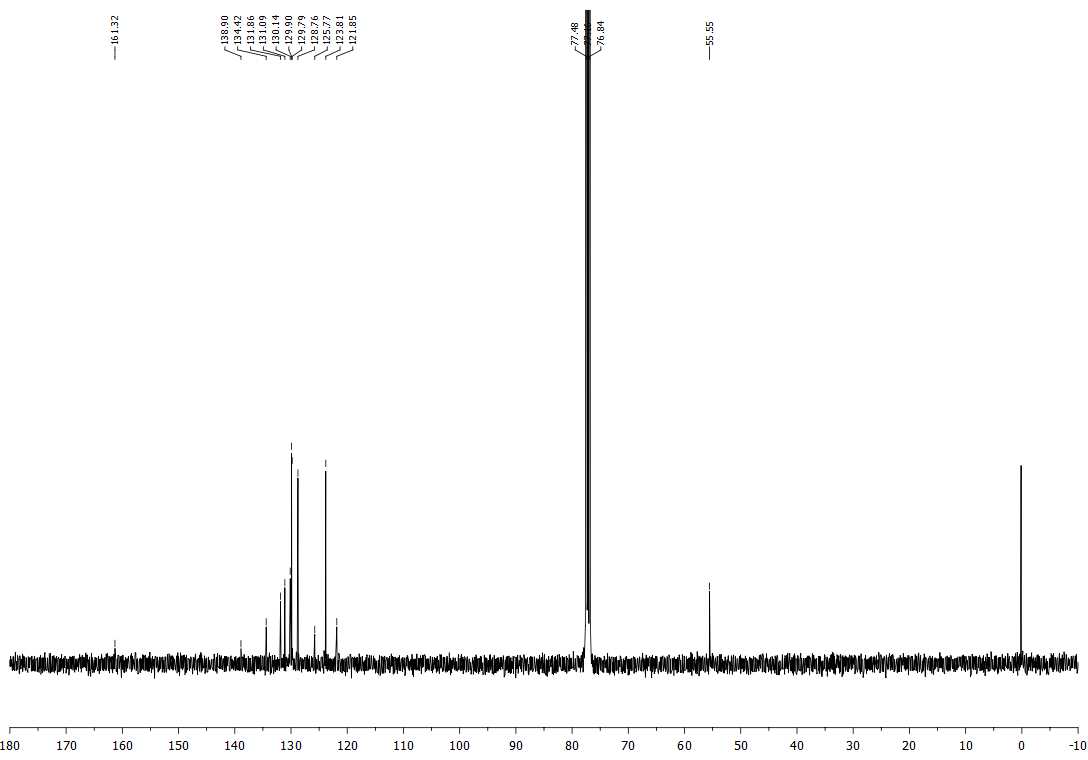
**

**13** (^1^H NMR, 400 MHz, CDCl_3_)


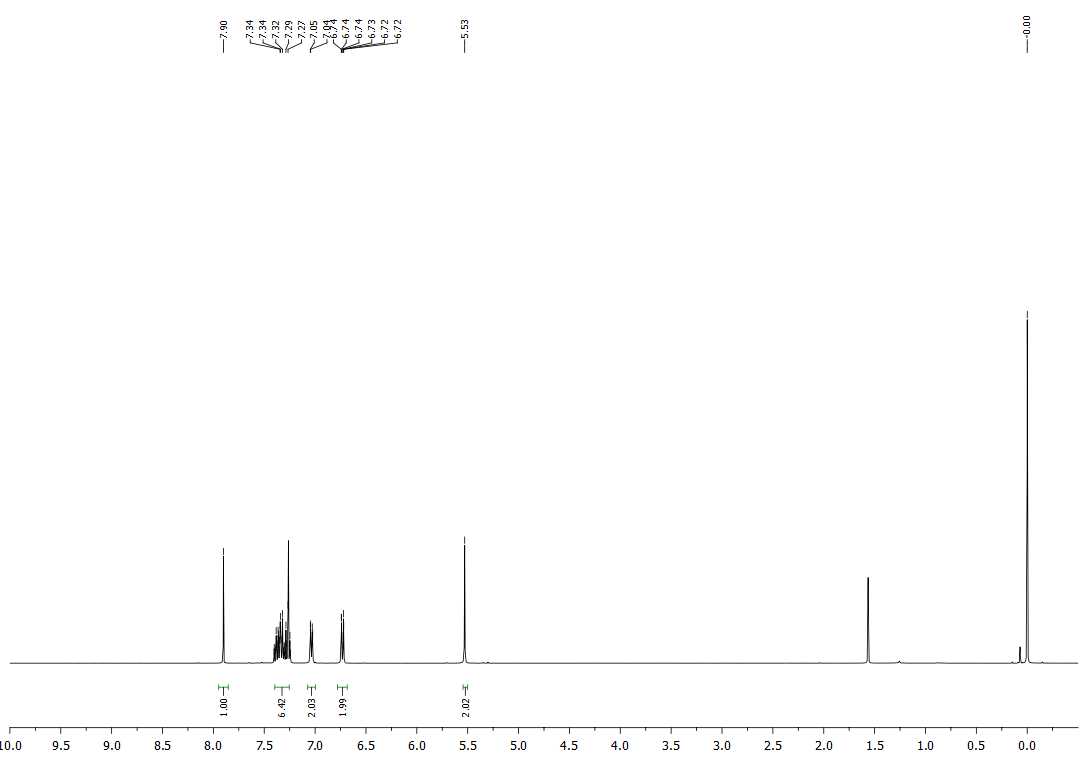


**13** (^13^C NMR, 75 MHz, CDCl_3_)


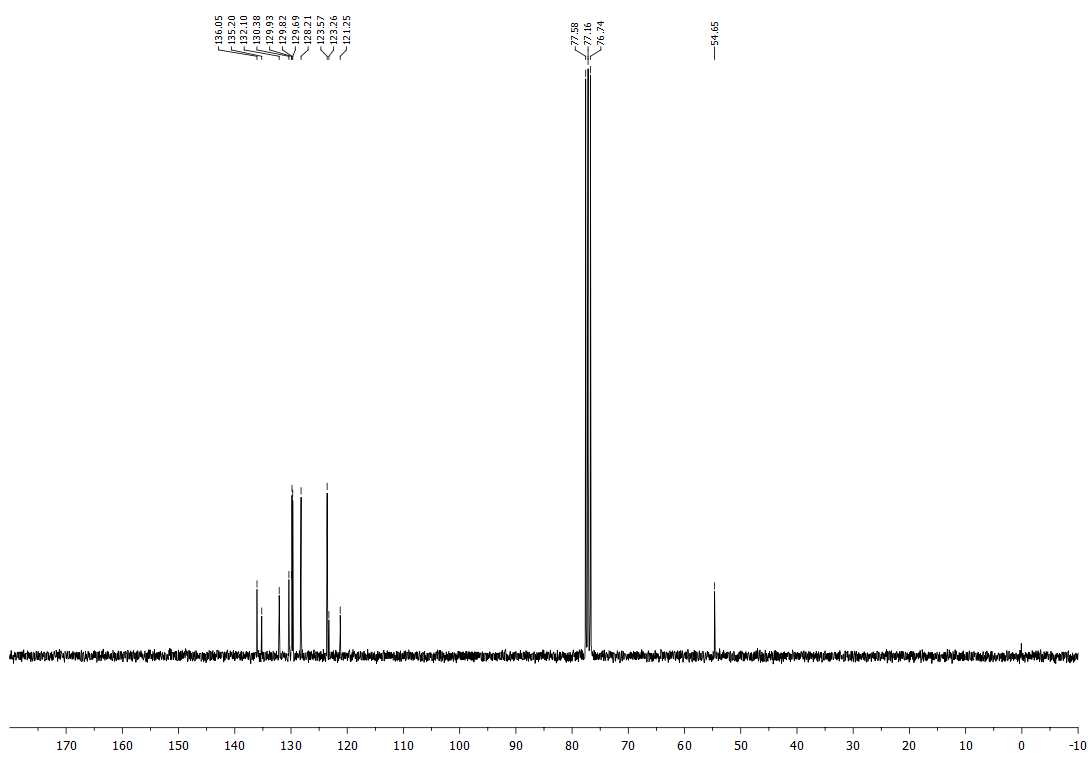


**14** (^1^H NMR, 400 MHz, CDCl_3_)


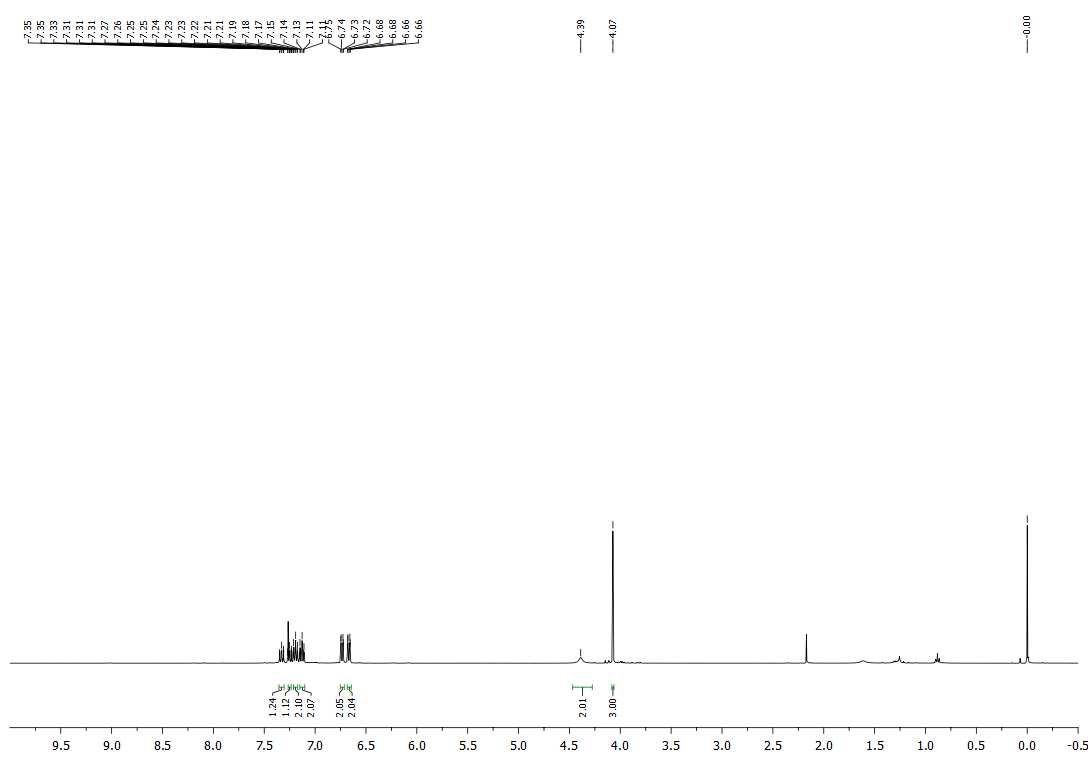


**14** (^13^C NMR, 75 MHz, CDCl_3_)


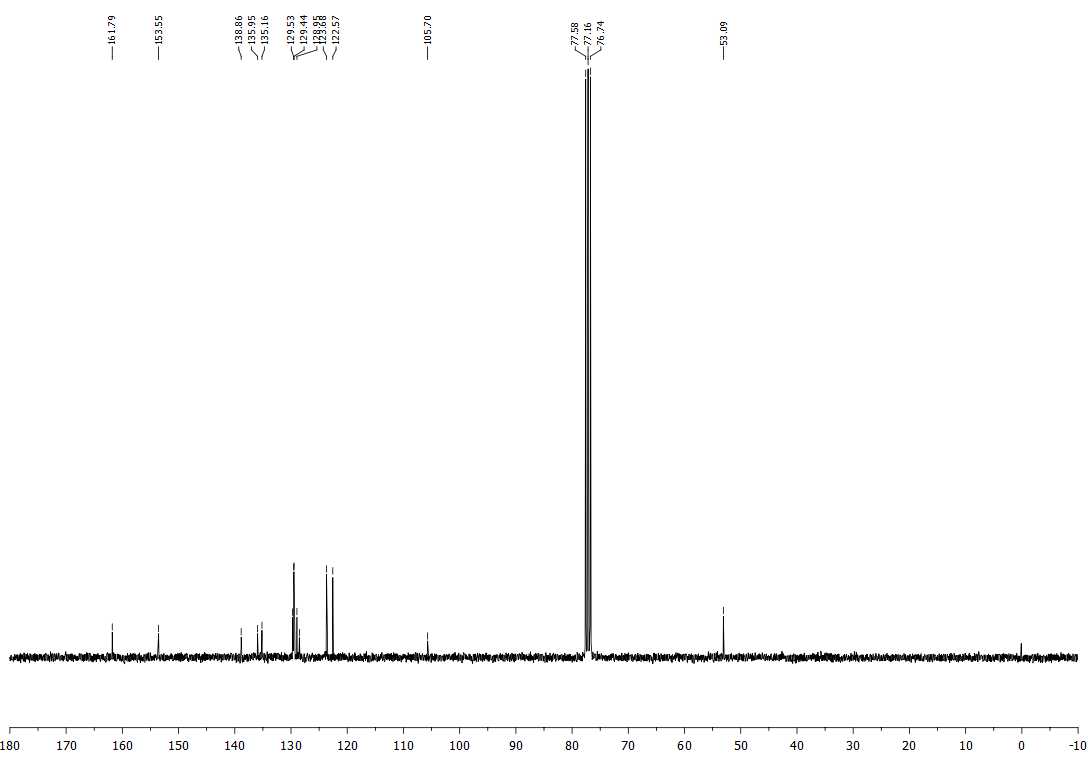


**15** (^1^H NMR, 400 MHz, DMSO-*d_6_*)


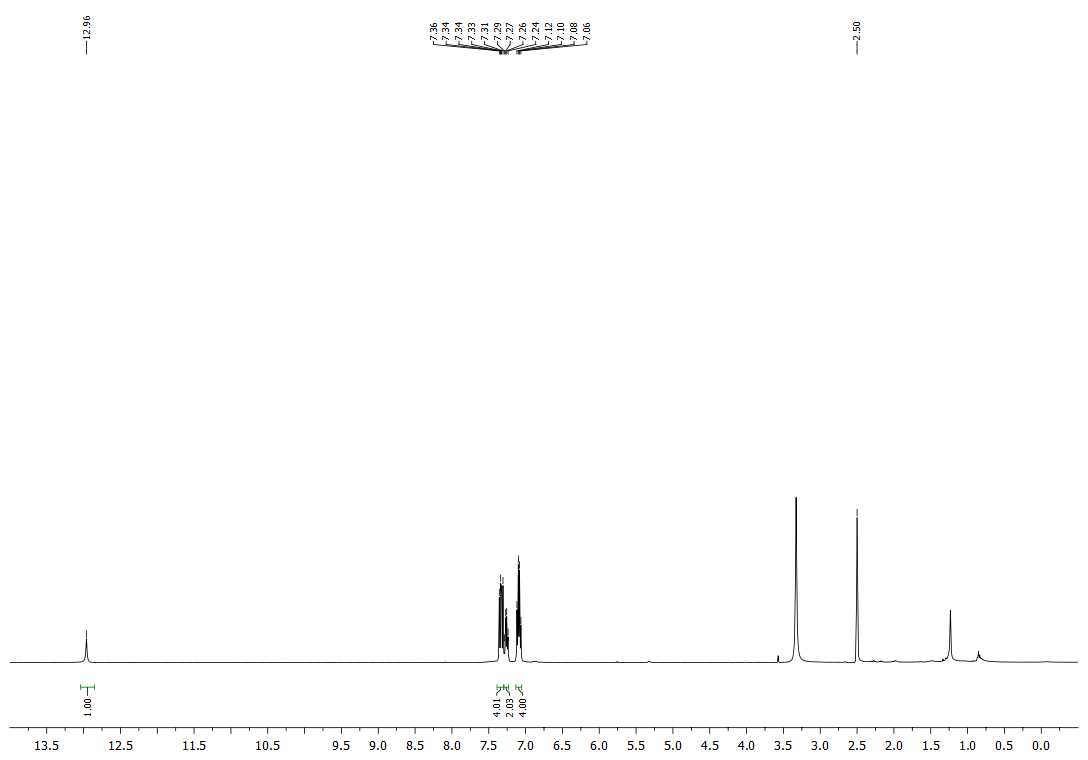


**15** (^13^C NMR, 101 MHz, DMSO-*d_6_*)


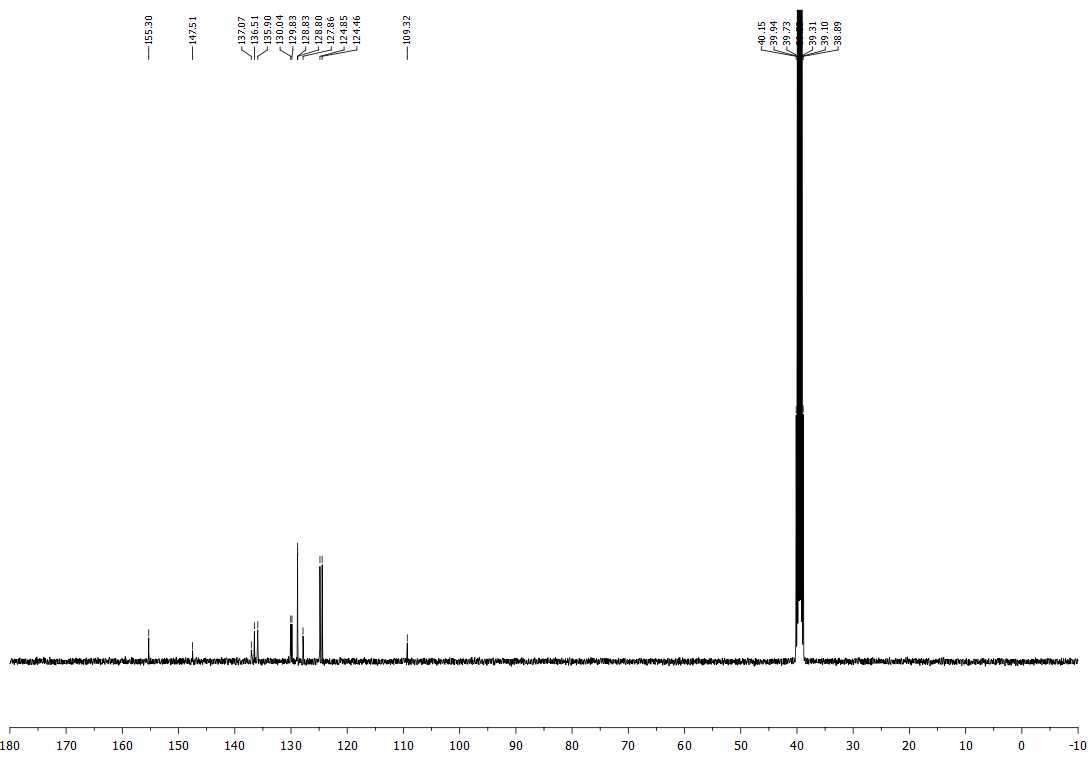


**References**

Campbell-Verduyn, L. S., Mirfeizi, L., Dierckx, R. A., Elsinga, P. H., and Feringa, B. L. (2009). Phosphoramidite accelerated copper(i)-catalyzed [3 + 2] cycloadditions of azides and alkynes. *Chem. Commun.*, 2139. doi:10.1039/b822994e.

Hu, M., Li, J., and Q. Yao, S. (2008). In Situ “Click” Assembly of Small Molecule Matrix Metalloprotease Inhibitors Containing Zinc-Chelating Groups. *Org. Lett.* 10, 5529–5531. doi:10.1021/ol802286g.

Maddani, M. R., Moorthy, S. K., and Prabhu, K. R. (2010). Chemoselective reduction of azides catalyzed by molybdenum xanthate by using phenylsilane as the hydride source. *Tetrahedron* 66, 329–333. doi:10.1016/j.tet.2009.10.093.

Thomas, J., John, J., Parekh, N., and Dehaen, W. (2014). A Metal-Free Three-Component Reaction for the Regioselective Synthesis of 1,4,5-Trisubstituted 1,2,3-Triazoles. *Angew. Chemie Int. Ed.* 53, 10155–10159. doi:10.1002/anie.201403453.

Titz, A., Radic, Z., Schwardt, O., and Ernst, B. (2006). A safe and convenient method for the preparation of triflyl azide, and its use in diazo transfer reactions to primary amines. *Tetrahedron Lett.* 47, 2383–2385. doi:10.1016/j.tetlet.2006.01.157.
